# Exploring the genetic diversity of Mediterranean fig trees highlights genes associated with fruit traits

**DOI:** 10.3389/fpls.2026.1750632

**Published:** 2026-02-10

**Authors:** Marco Castellacci, Gabriele Usai, Alberto Vangelisti, Samuel Simoni, Lucia Natali, Flavia Mascagni, Andrea Cavallini, Margarita Lopez-Corrales, Maria Guadalupe Domínguez, Ghada Baraket, Sahar Haffar, Ayzin Kuden, Songul Comlekcioglu, José Inaki Hormaza, Mitchell J. Feldmann, Steven J. Knapp, Tommaso Giordani

**Affiliations:** 1 Department of Agriculture, Food and Environment, University of Pisa, Pisa, Italy; 2 Área de Fruticultura Mediterránea, Instituto de Investigación Finca La Orden-Valdesequera (LA ORDEN-CICYTEX), Junta de Extremadura, Badajoz, Spain; 3 Department of Biology, Faculty of Sciences of Tunis, University of Tunis El Manar, Tunis, Tunisia; 4 Department of Horticulture, Faculty of Agriculture, Çukurova University, Adana, Türkiye; 5 Instituto de Hortofruticultura Subtropical y Mediterranea La Mayora, Agencia Estatal Consejo Superior de Investigaciones Cientificas, Málaga, Spain; 6 Department of Plant Sciences, University of California, Davis, Davis, CA, United States

**Keywords:** candidate genes, *Ficus carica* L, fruit quality traits, genomic resources, GWAS

## Abstract

The fig tree (*Ficus carica* L.) is a historically and economically important perennial crop in the Mediterranean region, valued for its fruit, leaves, and latex, which are employed in food, pharmaceutical, and cosmetic applications. Despite the agronomic and cultural relevance of *Ficus carica*, contemporary breeding programs are limited, and the genetic basis for major agro-morphological traits remains insufficiently characterised. To address this knowledge gap, we performed whole-genome resequencing on 286 genotypes from germplasm collections in Spain, Turkey, and Tunisia. Variant discovery identified 1,374,111 high-quality single nucleotide polymorphisms (SNPs), 2,448,766 small insertions/deletions, 218 copy number variants, and 1363 structural variants, many of which affect genes involved in stress responses, metabolism, signalling, and development. Population genomics revealed three main clusters corresponding to geographic origin, with some intermixing and cryptic relatedness reflecting historical germplasm exchange. By integrating genomic and phenotypic data, we identified 481 significant SNPs and candidate genes linked to 11 fruit traits and productive type, including genes associated with fruit weight (*FMO1* and *MYB* transcription factors), fruit size (*ABC* transporters and *WAK* kinases), firmness (*CML22* and sugar transporters *ERD6s*), sugar content and acidity (*CYP94C1* and *PPR* proteins), and productive type (*PP2C63*). This study represents the most comprehensive genomic and phenotypic resource for *F. carica* to date, providing a robust foundation for germplasm management, marker-assisted selection, and breeding strategies, including the application of genome editing technologies to accelerate the improvement of fruit quality, yield, and adaptability.

## 1 Introduction

The genus *Ficus* belongs to the Moraceae family, a group of angiosperms that comprises over 800 species worldwide. The most important species of the family is the fig tree (*Ficus carica* L.), which is native to the Middle East and has been domesticated in the Mediterranean region. The fig tree was one of the first plants cultivated by humans (Barolo et al., 2014). Its current distribution, fruit morphology, and genetic diversity have been shaped by its domestication history and subsequent worldwide expansion (Ayuso et al., 2022). Annually, over one million tons of fig fruit are produced worldwide, with about 90% of production concentrated in Turkey, Egypt, Morocco, Algeria, Spain, the USA, and Tunisia. Turkey is the leading producer, harvesting 356,000 tons (FAO, 2023).

*Ficus carica* is a versatile perennial species used in various forms in the food, health, and cosmetic industries. Its fruit, both fresh and dried, is commonly processed into products, such as jams, preserves, jellies, and juices, making it a staple ingredient in traditional and modern diets (Khoshnoudi-Nia et al., 2023). The leaves also have significant value in traditional medicine and are often prepared as decoctions or infusions for therapeutic use (Morovati et al., 2022; Rasool et al., 2023). Additionally, the latex and other plant parts are applied in the formulation of cosmetic and pharmaceutical products, highlighting the multifaceted utility of this plant (Badgujar et al., 2014; Alzahrani et al., 2024). Furthermore, fig plants exhibit moderate tolerance to environmental stressors, such as drought and salinity, highlighting their relevance in climate change and sustainable agriculture (Vangelisti et al., 2019).

Although *F. carica* has been cultivated for centuries, it has not been subjected to extensive modern breeding efforts. Many fig varieties originated from the selection of seed-derived plants and were subsequently spread and maintained through vegetative propagation and local adaptation (Mazzeo et al., 2024). As a result, many fig populations (i.e. groups of genotypes that are naturally or traditionally grown in the same geographic area) have retained a high degree of genetic diversity, which holds great potential but remains underutilised until properly identified and categorised. Traditionally, the characterisation of fig germplasm for conservation purposes has relied on morphological and agronomic traits. However, conventional methods are limited by interannual, environmental, and replication variability, hindering consistent and reliable classification (Khadivi and Mirheidari, 2023). Molecular markers based on DNA polymorphisms, such as simple sequence repeats (SSRs) have been used recently (Giraldo et al., 2005; Perez-Jiménez et al., 2012; Hssaini et al., 2020); however, resolution was often low. As a matter of fact, a notable degree of ambiguity and inconsistency exists in the naming and identification of cultivated varieties across different regions and countries (Perez-Jiménez et al., 2012; Ergül et al., 2021; Bazakos et al., 2024).

To address these challenges, new molecular tools have emerged as powerful alternatives for adequately characterising fig tree varieties. Advances in next-generation sequencing have revolutionised genotyping capabilities (Zahid et al., 2022). Among these, whole-genome resequencing (WGR) has expanded rapidly, driven by declining DNA sequencing costs, and many plant species have already been sequenced (Song et al., 2023). WGR consists of sequencing the genomes of specific individuals or populations using an existing reference genome to uncover variations, including single nucleotide polymorphisms (SNPs), small insertions and deletions (InDels), copy number variants (CNVs), and structural variants (SVs). These data enable molecular genetic analyses of populations, studies on genetic evolution, and the identification of genes linked to key traits through genome-wide association studies (GWASs) (Li et al., 2023; Song et al., 2024), which have been performed on many fruit species (Cao et al., 2016; Ferik et al., 2022; Zheng et al., 2022; Holušová et al., 2023; Dujak et al., 2024; Bazakos et al., 2024).

In this study, we applied WGR to 286 fig genotypes from diverse germplasm collections and geographic origins of the Mediterranean region to identify genome-wide variations. For this purpose, fig genotypes were resequenced using the most recent version of the *F. carica* ‘Dottato’ genome as a reference. This genome is a comprehensive and well-annotated assembly developed using PacBio sequencing and Hi-C mapping strategies (Usai et al., 2025). Moreover, GWAS was performed to identify specific genetic regions and potential candidate genes associated with market-valued traits, such as fruit weight (WE), length (LG), firmness (FM), and juiciness (FJ). Compared to previous studies (Ergül et al., 2021; Essid et al., 2021; Bazakos et al., 2024; Radunić et al., 2025), our study is distinguished by the most extensive and diverse panel of fig genotypes analysed by GWAS, the use of the most recent high-quality reference genome to identify variants, and the comprehensive quantitative phenotyping of market-relevant traits. In conclusion, to our knowledge, this study represents the most comprehensive effort to evaluate the genetic variability of cultivated fig trees and offers a valuable resource for applying modern molecular procedures to the genetic improvement of this fruit species.

## 2 Materials and methods

### 2.1 Plant material

A total of 286 fig varieties, comprised of 18 caprifigs and 268 female varieties, from 3 germplasm banks in Spain (61 genotypes), Turkey (115), and Tunisia (110) were studied (**Supplementary Table S1**). The Spanish fig tree germplasm bank is at the Institute of Research ‘Finca La Orden’ (Guadajira, Badajoz, Spain) and belongs to CICYTEX (Scientific and Technological Research Centre of Extremadura, Spain). The 61 genotypes included in the Spanish germplasm collection originated from Spanish regions, including Extremadura, Catalonia, Balearic Islands, Andalusia, Castile-La Mancha, Canary Islands, and Castilla, and Leon, and the Valencian Community, as well as from Turkey, Israel, and Ethiopia (**Supplementary Table S1**).

The Turkish fig germplasm bank is at the Fig Research Institute in Erbeyli/Aydin region. The 115 genotypes selected from this collection came from various regions of Turkey, such as the Black Sea, Mediterranean, Aegean, Southeast and Central Anatolia, and Marmara, and one genotype was from Israel (**Supplementary Table S1**).

Of the 110 genotypes in the Tunisian germplasm collection, 104 were sourced from the Arid Regions Institute (IRA) in Mednine, the Regional Center for Oasis Agriculture Research (CRRAO) in Degache, the Higher Institute of Agronomy (ISA) in Chott Meriem, and the Regional Commissary for Agricultural Development (CRDA) in Gafsa, Seliana, and Gabes. The remaining six genotypes were collected from Utique, Menzel Hbib, and the Sahel region. All Tunisian genotypes originated from diverse areas, such as Gafsa, Degache, Utique, Kesra, Djebba, Bir Amir, and Kerkennah Island (**Supplementary Table S1**).

### 2.2 Whole genome re-sequencing

Young leaves were collected from the 286 fig genotypes, and the genomic DNA was extracted using the Macherey-Nagel™ NucleoSpin™ Plant II Kit, according to the manufacturer’s instructions. DNA was quantified using a Qubit 2.0 fluorometer (Invitrogen, Carlsbad, CA, USA) and its quality was tested using an Agilent 2100 Bioanalyzer High Sensitivity DNA Assay (Agilent Technologies, Santa Clara, CA, USA). DNA libraries were built using a CeleroTM DNA-Seq Library Preparation Kit (Tecan Genomics, Redwood City, CA, USA) according to the manufacturer’s instructions. DNA libraries were sequenced using an Illumina NovaSeq 6000 in paired-end 150-bp mode.

### 2.3 Variant calling, filtering, and annotation

FastQC v0.11.9 (Andrews, 2010) was used to process sequence quality checks of the FASTQ-formatted read packages. To enhance the quality and precision of subsequent analyses, read datasets underwent cleanup using Trimmomatic v0.39 (Bolger et al., 2014), followed by thorough quality control assessment.

All reads were mapped against the reference fig genome downloaded from the National Center for Biotechnology Information under Bioproject PRJNA1111048 (Usai et al., 2025) using BWA software v0.7.17 (Li and Durbin, 2009). The MarkDuplicates function in Picard tool v2.26.2 (Picard toolkit, 2019) was used to locate and remove duplicate reads in each BAM file.

SNPs and InDels were called using HaplotypeCaller, Combine GVCFs, and GenotypeGVCFs functions in GATK v4.2.6.1 (Van der Auwera and O’Connor, 2020).

Hard filtering for SNPs was performed using GATK v4.2.6.1 (Van der Auwera and O’Connor, 2020). SNPs were filtered with the following criteria: --filter-name ‘QD2’ --filter-expression ‘QD < 2.0’ --filter-name ‘QUAL30’ --filter-expression ‘QUAL < 30.0’ --filter-name ‘SOR3’ --filter-expression ‘SOR > 3.0’ --filter-name ‘FS60’ --filter-expression ‘FS > 60.0’ --filter-name ‘MQ40’ --filter-expression ‘MQ < 40.0’ --filter-name ‘MQRankSum-12.5’ --filter-expression ‘MQRankSum < −12.5’ --filter-name ‘ReadPosRankSum-8’ --filter-expression ‘ReadPosRankSum < −8.0’.

SNPs with a missing rate > 20% and minor allele frequency (MAF) less than 0.05 were removed. Genotype imputation was performed using Beagle v5.4 (Browning et al., 2021).

InDel variants were filtered using GATK v4.2.6.1 (Van der Auwera and O’Connor, 2020) with the following criteria: --filter-name ‘QD2’ --filter-expression ‘QD < 2.0’ --filter-name ‘QUAL30’ --filter-expression ‘QUAL < 30.0’ --filter-name ‘FS200’ --filter-expression ‘FS > 200.0’ --filter-name ‘ReadPosRankSum-20’ --filter-expression ‘ReadPosRankSum < −20.0’ --filter-name ‘ExcessHet-54.69’ --filter-expression ‘ExcessHet > 54.69’ --genotype-filter-name ‘GQ-30’ --genotype-filter-expression ‘GQ < 30’ --genotype-filter-name ‘DP-4and3000’ --genotype-filter-expression ‘DP < 3000 || DP > 4’.

High-quality SNPs and InDels were annotated using SNPeff v5.3 (Cingolani et al., 2012).

### 2.4 Structural variant calling and filtering

Genome-wide CNVs (for sequences > 1 kb in length) were analysed using control-FREEC v11.6 (Boeva et al., 2012). Statistical significance was evaluated using assess_significance, an R script that computes p-values based on both the Wilcoxon and Kolmogorov–Smirnov tests. For downstream analysis, we applied a stringent filtering threshold, retaining only CNVs with a Kolmogorov–Smirnov *p*-value ≤ 0.05. Variants located less than 100 bp apart were merged, and mixed-type sequences (showing both gains and losses) and those present in fewer than three genotypes were excluded to enhance data quality.

SVs (for sequences > 50 bp) for each genotype were identified using three programs, namely Manta v1.6.0 (Chen et al., 2016), LUMPY v0.2.13 (Layer et al., 2014), and DELLY v0.8.1 (Rausch et al., 2012), executed with default parameters. The resulting VCF files for individual genotypes were consolidated into a single file using SURVIVOR v1.07 (Jeffares et al., 2017). Variants were filtered based on specific criteria: MAF of 0.01, SV less than 1 Mbp, and no more than 40% missing genotype data.

Two binary tables were generated to represent the presence or absence of CNVs and SVs across the 286 genotypes. Each entry in these tables was coded as “1” to indicate presence and “0” to indicate absence, offering a clear framework for comparison and visualisation.

Gene Ontology (GO) term enrichment of genes containing CNVs and SVs was performed using Blast2GO v5.2.5 (Conesa et al., 2005).

### 2.5 Population structure and kinship analysis

To perform population structure analysis, SNPs were filtered using Plink v1.90 (Purcell et al., 2007) with the criteria “–indep-pairwise 50 5 0.2”. The population structure of 286 genotypes was examined using STRUCTURE software v2.3.4 (Pritchard et al., 2000), considering K values from K = 1 to K = 6, and principal component analysis (PCA) was conducted using the SNPrelate package (Zheng et al., 2012) in R v4.2.2 (R Core Team, 2022).

A maximum likelihood phylogenetic tree was built using the Snphylo pipeline (Lee et al., 2014). In this case, the whole SNP dataset was pruned using a sliding window of 500,000 nt and an r^2^ value of 0.1. Default parameters were applied in the pipeline, and 1000 bootstrap replicates were performed to generate a bootstrapped maximum likelihood tree.

To estimate the genetic diversity between and within germplasm banks, we calculated the population differentiation (fixation index, FST), nucleotide diversity (π), observed heterozygosity (Ho), and expected heterozygosity (He) using VCFtools v0.1.16 (Danecek et al., 2011).

SVs data were used to investigate population structure based on a presence-absence matrix. PCA was performed using the R package FactoMinerR (Lê et al., 2008), and the R package pvclust version 2.2-0 (Suzuki and Shimodaira, 2006) was used to build a dendrogram on the resulting Jaccard distance matrix.

Kinship analysis was performed using KING v2.3.0 (Manichaikul et al., 2010) with the “-kinship” option. The analysis was performed using the complete set of unpruned SNPs as input. The estimated kinship coefficients were visualised as a network, representing potential genetic relationships among the genotypes. The network was visualised using the ggnet2 function from the GGally package (Schloerke et al., 2021) in R.

### 2.6 Morphological characterisation and statistical analysis

Of 286 fig genotypes, 257 were characterised for morphological and pomological traits (**Supplementary Table S13**). All caprifigs and a few female figs were excluded from phenotyping. The phenotypic observations of 23 fig traits were conducted for 2 consecutive years (2021–2022).

Plant and fruit traits were determined according to the fig tree descriptors (TG/265/1) of the International Union for the Protection of New Varieties of Plants (UPOV) guidelines (UPOV, 2010).

Fruit traits, such as weight, size, total soluble solid content (TSS), titratable acidity (TA), and firmness (FM) were measured using 10 randomly selected fig fruits per genotype.

Weight (g) was determined using a Mettler AE-166 balance (Mettler-Toledo, Greifensee, Switzerland). The size, related to fruit dimensions, was determined using a digital calliper measuring the length and width of the fruit, including the ostiole size and the stalk length. To calculate the TSS and TA, some fruit was peeled and homogenised in a blender. The TSS, expressed as °Brix, was determined with an RM40 digital refractometer (Mettler-Toledo, Greifensee, Switzerland). The TA, expressed as citric acid g/100 g fresh weight, was determined with a T50 automatic titrator (Mettler-Toledo, Greifensee, Switzerland). Aliquots of 5 g of the homogenised samples were diluted in 50 mL deionised water and titrated with 0.1 mol/L NaOH up to pH 7.8 (Serrano et al., 2005). The FM was measured with a TA-XT Texture Analyser (Stable Micro Systems Ltd, Godalming, United Kingdom) applying a force to produce a 6% deformation with a 70 mm aluminium plate. The slope was determined in the linear zone of the force-deformation curve, and the results are expressed as N/mm.

To ensure a thorough analysis of our data, phenotypic traits were analysed using a linear mixed model in R package lme4::lmer (Bates et al., 2015). For quantitative traits, repeatability (R) was calculated from the resulting phenotypic variance components, with genotype and location included as random effects across two years (**Supplementary Table S14**). Ordinal and categorical traits were excluded from repeatability analyses due to violations of model assumptions. Estimated marginal means (EMMs) for each genotype, trait, and year were calculated using the R package emmeans (Lenth et al., 2021) to provide phenotypic values for GWAS (**Supplementary Table S15**).

### 2.7 Genome-wide association study

A multi-trait association analysis was performed based on the identified SNPs using GEMMA software v0.98.1 (Zhou and Stephens, 2012). For each phenotypic trait, the EMMs from two years of phenotyping were treated as individual traits (**Supplementary Table S15**). To adjust for population structure effects, a genetic relationship matrix was constructed using the whole SNP dataset and the same program.

To detect significant SNPs associated with traits, for each GWAS result, we calculated the Bonferroni correction as described by Kaler and Purcell (2019). As a graphical representation of the GWAS results for each trait, Manhattan plots and QQ-plots were generated using the qqman package (Turner, 2018) in R. The genomic inflation factor (λ) was calculated in R as the ratio of the median observed χ² statistic to the expected median under the null, using p-values from GWAS results.

## 3 Results

### 3.1 Exploring genomic variation in fig trees

**Figure 1:**
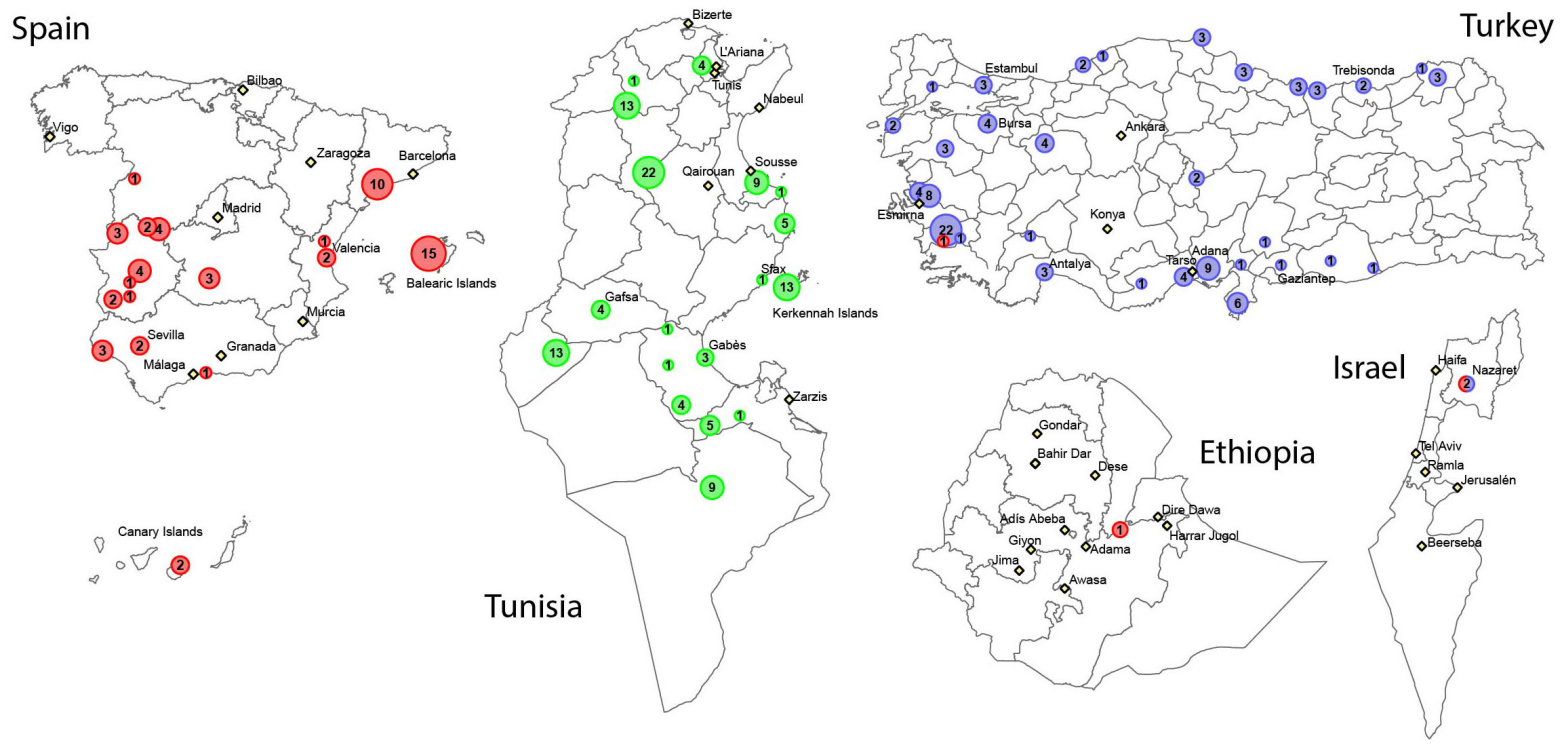
Geographical origin of *Ficus carica* genotypes analysed in this study (the numbers in the circles indicate the number of genotypes from a specific region). Genotypes in red, blue, and green are from the Spanish, Turkish, and Tunisian germplasm collections, respectively.

In this study, we analysed 286 fig genotypes originating from diverse germplasm collections and geographic areas across the Mediterranean region to identify and characterise genome-wide variations. The geographic distribution and germplasm collection origins of these genotypes are shown in **Figure 1**.

**Figure 2:**
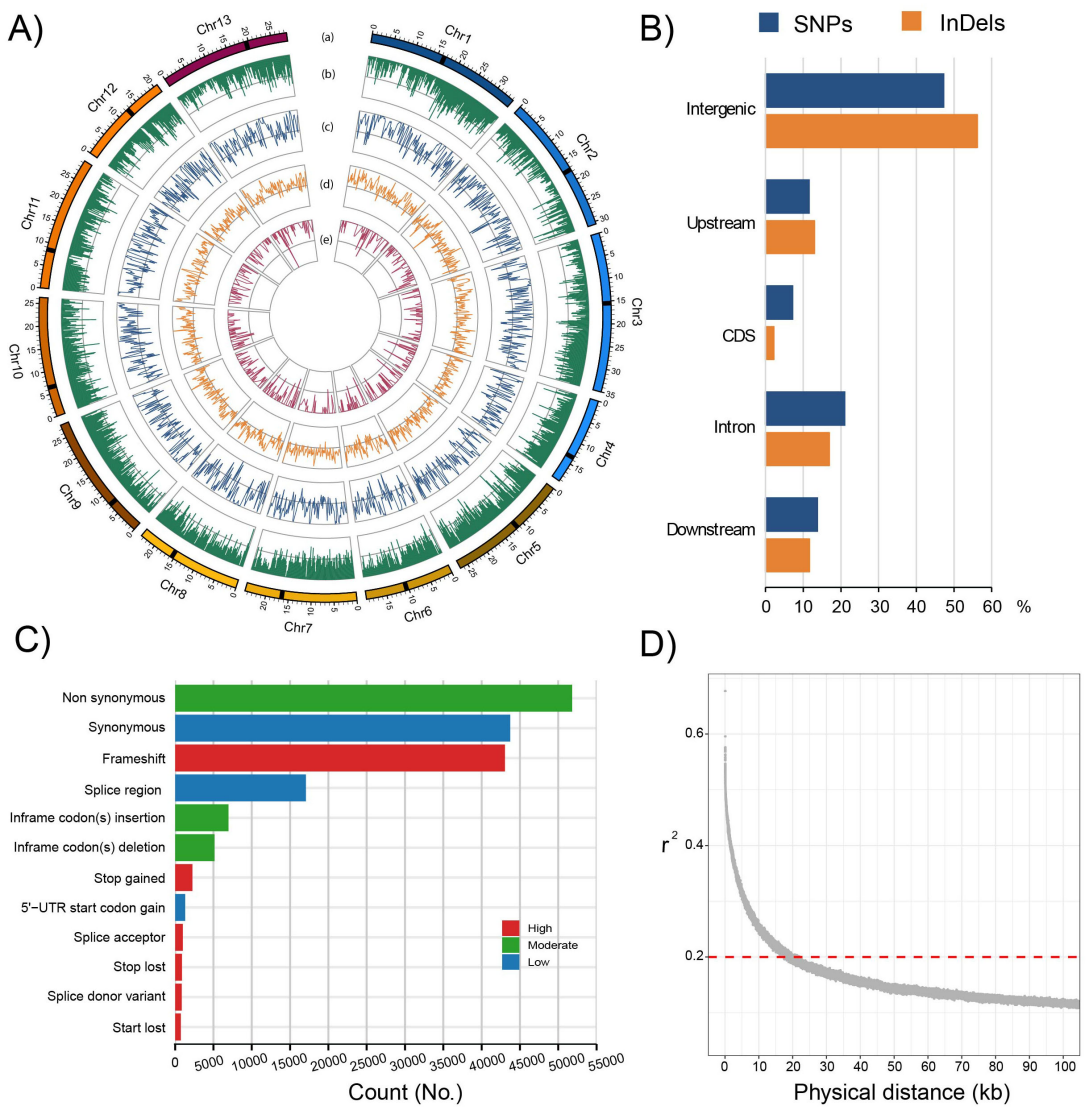
**(A)** Circular plot showing the 13 chromosomes of *Ficus carica*. From the innermost to the outermost rings: the 13 chromosomes (a), gene density (b), SNP density (c), InDel density (d), and SV density (e). **(B)** Distribution of SNPs and InDels across genic and intergenic regions. **(C)** Functional classification of SNPs and InDels in coding sequences according to their predicted effects. **(D)** Genome-wide linkage disequilibrium (LD) decay, measured as the decline r^2^ with increasing physical distance between SNPs.

WGR of 286 fig genotypes generated 846 GB of data, resulting in an average coverage of 18.31× (median: 17.35×; range: 12.07×–57.46×). After quality filtering, clean reads were mapped against the latest version of the fig reference genome (Usai et al., 2025) (**Supplementary Table S2**). Mapping results were used to identify SNPs, InDels (< 50 bp), CNVs, and SVs (> 50 bp). The genome-wide distribution of these variants is shown in **Figure 2A**.

Through variant calling, SNP filtering, and imputation, a final dataset was produced from the 286 genotypes, uncovering 1,374,111 high-quality SNPs, with a SNP density of 1 SNP every 252 bases.

Most SNPs were spread across the genome (**Supplementary Table S3**), with the majority in intergenic regions (47.11%). Over 700,000 SNPs were identified in genic regions. The majority were located in introns (20.85%), followed by 13.60 and 11.41% within 1000 nt downstream and upstream coding regions, respectively (**Figure 2B**). A total of 96,726 SNPs (7.04%) were detected in coding regions (**Figure 2B**). In the coding sequences, we predicted the putative effects on gene function, with high, moderate, and low impact (**Figure 2C**). We identified 51,808 non-synonymous and 43,723 synonymous mutations (**Figure 2C**). The ratio of non-synonymous to synonymous SNPs (K_a_/K_s_) was 1.2.

A total of 2,448,766 InDels, comprising 1,270,023 insertions and 1,178,743 deletions, were observed (**Supplementary Table S4**). Single nucleotide InDels were the most common (53.64%), followed by dinucleotide (14.99%) and trinucleotide InDels (6.72%). Most InDels were identified in intergenic regions (56.19%), followed by introns (16.93%) and within 1000 nt upstream (12.98%) and downstream (11.66%) of coding sequences. In the coding regions, 2.25% of InDels were detected (**Figure 2B**). Annotation and the putative impact of these variants are shown in **Figure 2C**.

The genome-wide diversity and linkage disequilibrium (LD) analysis revealed a relatively rapid LD decay, with r² ≤ 0.2 observed at approximately 18 kb in the analysed genotypes (**Figure 2D**).

**Figure 3:**
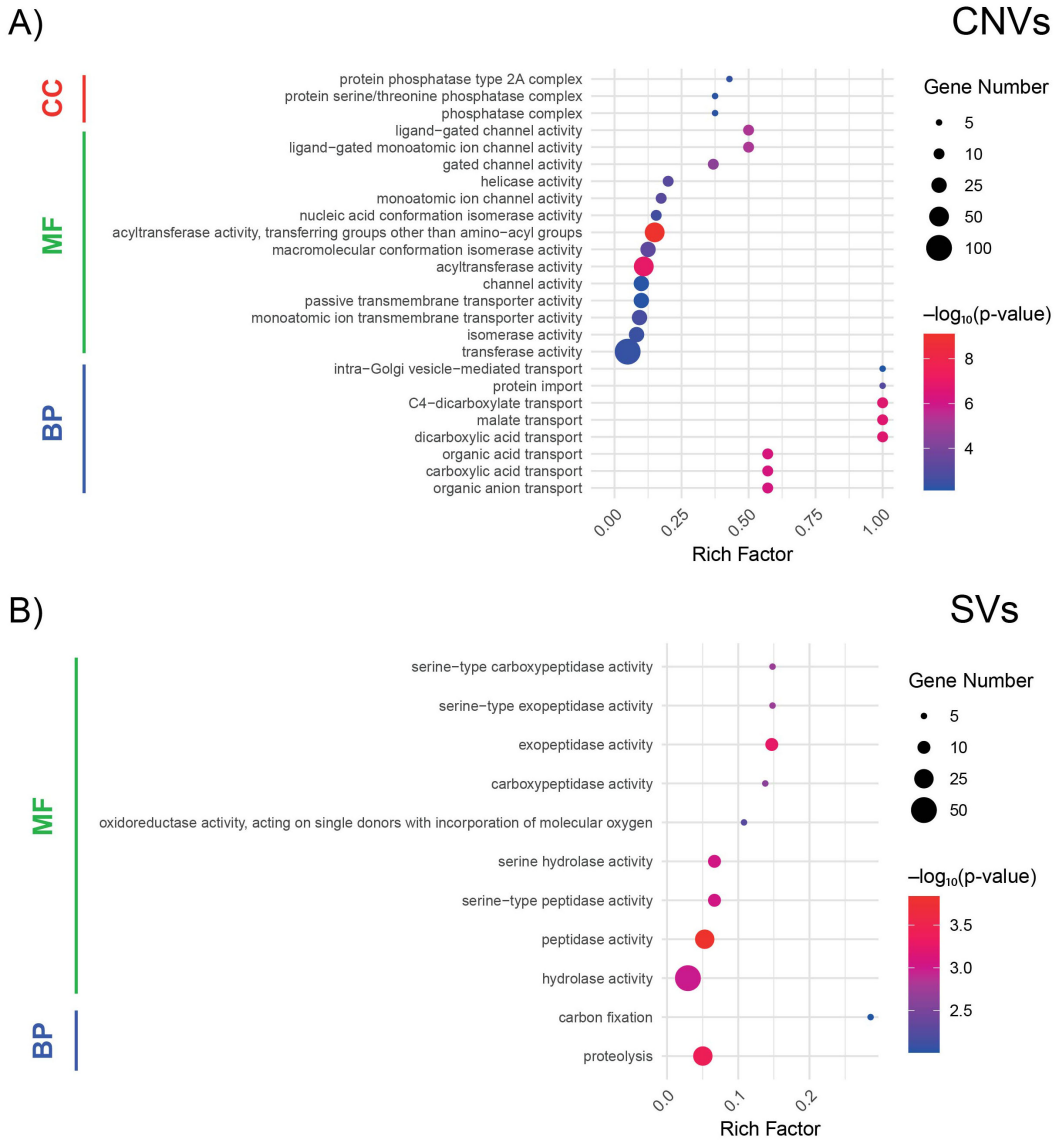
Functional enrichment analysis of genes affected by copy number variations (CNVs) and structural variants (SVs). **(A)** Gene Ontology (GO) enrichment analysis for CNV-associated genes. Terms are grouped into Cellular Component (CC, red), Molecular Function (MF, green), and Biological Process (BP, blue). The x-axis represents the Rich Factor. Dot size corresponds to the number of genes, and dot color indicates statistical significance (−log_10_(p-value)). **(B)** GO enrichment analysis for SV-associated genes using the same color and size scheme. Enriched terms highlight the molecular functions and biological processes most affected by these genomic variants.

Genome-wide CNV analysis revealed the presence of 218 CNVs, divided into 94 copy number gains (CNGs) and 121 copy number losses (CNLs), with an average of 0.76 CNVs per genotype(**Supplementary Table S5**). Of the CNVs, 154 were present in a maximum of two genotypes, with most of them being genotype specific. In contrast, the other half of the variants were distributed across a range of individuals (**Supplementary Figure 1**). The number of CNLs was higher than the number of CNGs on chromosomes 1, 3, 4, 7, 9, 10, and 13, whereas for chromosomes 2, 6, 8, 11, and 12, the number of CNGs was higher than that of CNLs (**Supplementary Figure 2**). Chromosome 5 showed a similar number of CNL and CNG variants. The distribution of CNVs covered the largest portion of the chromosomes (**Supplementary Figure 3**). Of the identified CNVs, 49 CNGs and 107 CNLs were located within 1227 genes of the fig tree genome (**Supplementary Tables 6**, **7**). In particular, the most frequently enriched CNV-associated GO terms (**Figure 3A**) within the Biological Process category were ‘intra-Golgi vesicle mediated transport’ (GO:0006891), ‘protein import’ (GO:0017038), and ‘C4-dicarboxylate transport’ (GO:0015740). Within the molecular Function category, the top enriched terms included ‘ligand-gated channel activity’ (GO:0022834), ‘ligand-gated monoatomic ion channel activity’ (GO:0015276), and ‘gated channel activity’ (GO:0022836). Finally, within the Cellular Component category, the most enriched terms were ‘protein phosphatase type 2A complex’ (GO:0000159), ‘protein serine/threonine phosphatase complex’ (GO:0008287), and ‘phosphatase complex’ (GO:1903293) (**Supplementary Table S8**).

Additionally, SV calling was performed. In total, we detected 1363 SVs, comprising 1342 deletions and 21 duplications, corresponding to an average of 4.76 SVs per genotype (**Supplementary Table S9**). Of these, 318 SVs occurred in 606 genes. The distribution of deletions was uniform across the 13 chromosomes, and each chromosome had at least one duplication variant, except chromosomes 1, 6, 7, and 11 (**Supplementary Figure 4**). Among the 606 genes affected by SVs, a wide array of functional categories emerged, highlighting the diverse roles these genes may play in fig tree biology (**Supplementary Table S10**). For SVs, the most frequently enriched GO terms (**Figure 3B**) within the Molecular Function category were ‘serine-type carboxypeptidase activity’ (GO:0004185), ‘serine-type exopeptidase activity’ (GO:0070008), and ‘exopeptidase activity’ (GO:0008238). Within the Biological Process category, enrichment was observed for ‘carbon fixation’ (GO:0015977) and ‘proteolysis’ (GO:0006508) (**Supplementary Table S11**).

### 3.2 Population structure analysis

The population structure of the 286 fig tree genotypes was analysed by performing principal component analysis, model-based clustering, and constructing a phylogenetic tree.

**Figure 4:**
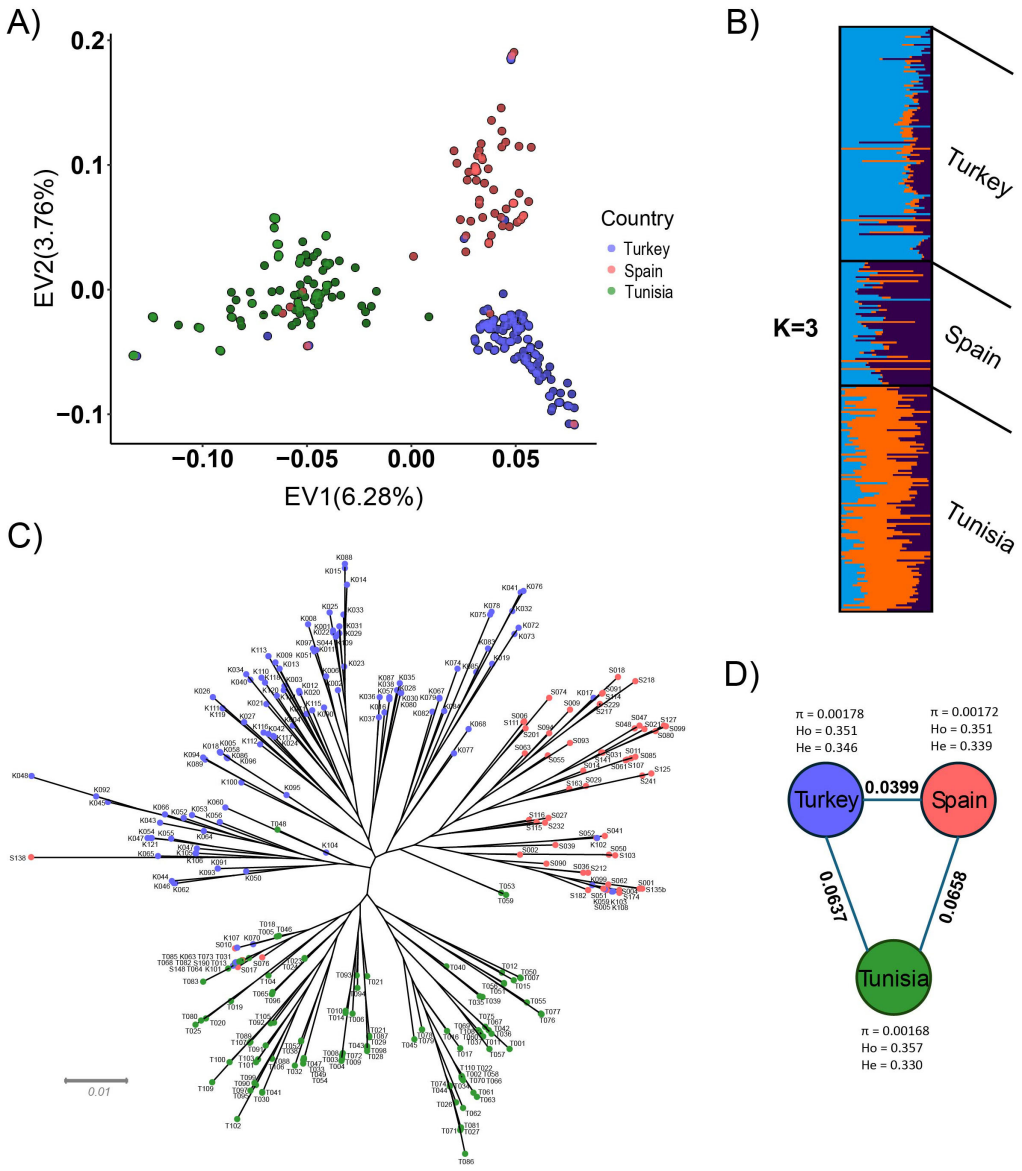
Genetic diversity of 286 fig tree genotypes. Genotypes from the Spanish, Turkish and Tunisian collections are shown in red, blue, and green, respectively. **(A)** Principal component analysis (PCA) showing the projection of the first two axes. **(B)** Population structure analysis at K = 3 showing the inferred genetic clusters. **(C)** Neighbor-joining dendrogram, with the grey scale bar indicating genetic distance. **(D)** Graphic representation of genetic distance among three germplasm banks using FST values represented on each line, nucleotide diversity (π), expected heterozygosity (H_e_), and observed heterozygosity (H_o_).

SNP pruning resulted in 54,989 SNPs, which were used for PCA and structure analysis. The first and second components of PCA accounted for 6.28 and 3.76% of the genetic variation, respectively, revealing three major genotype clusters that largely corresponding to the country of origin (**Figure 4A**). Structure analysis through the Evanno method showed that the best number of K was three (**Figure 4B**). In particular, **Figure 4B** shows that these three ancestries contributed differently to genetic structure in genotypes from different countries, confirming the PCA results. Most Turkish genotypes were clearly distinguished, as they were the most abundant light blue component, and in the Spanish ones, the purple component was predominant. In the Tunisian genotypes, the orange component was the most represented.

However, there was evidence of intermixing, with some genotypes from Turkey, Spain, and Tunisia appearing in clusters associated with other countries, as also shown by the phylogenetic tree (**Figure 4C**). This pattern likely reflects the historical exchange of germplasm and the reintroduction of varieties under different local names. For instance, two Spanish genotypes were found in the Turkish cluster. In addition, six Turkish genotypes clustered with Spanish samples, and four Turkish and five Spanish genotypes were grouped with Tunisian genotypes.

We evaluated the genetic diversity (**Figure 4D**) between each germplasm bank using the FST. The FST values of the three groups ranged from 0.0399 to 0.0658. The expected heterozygosity (H_e_) of *F. carica* populations was between 0.330 and 0.346, and the observed heterozygosity (H_o_) was between 0.351 and 0.357. The highest mean nucleotide diversity (π) was observed in the Turkish collection (0.00178), followed by the Spanish (0.00172) and Tunisian collections (0.00168).

Structural variants (SVs) were further employed to investigate genetic diversity among fig genotypes (**Supplementary Figure 5**). PCA clearly separated the Tunisian genotypes, whereas Spanish and Turkish samples exhibited substantial overlap. Nevertheless, phylogenetic analysis supported the presence of three distinct populations. Conversely, PCA did not separate Tunisian, Spanish, and Turkish genotypes based on copy number variants (CNVs, data not reported) probably due to their very low frequency in the population (0.76 CNVs per genotype).

**Figure 5:**
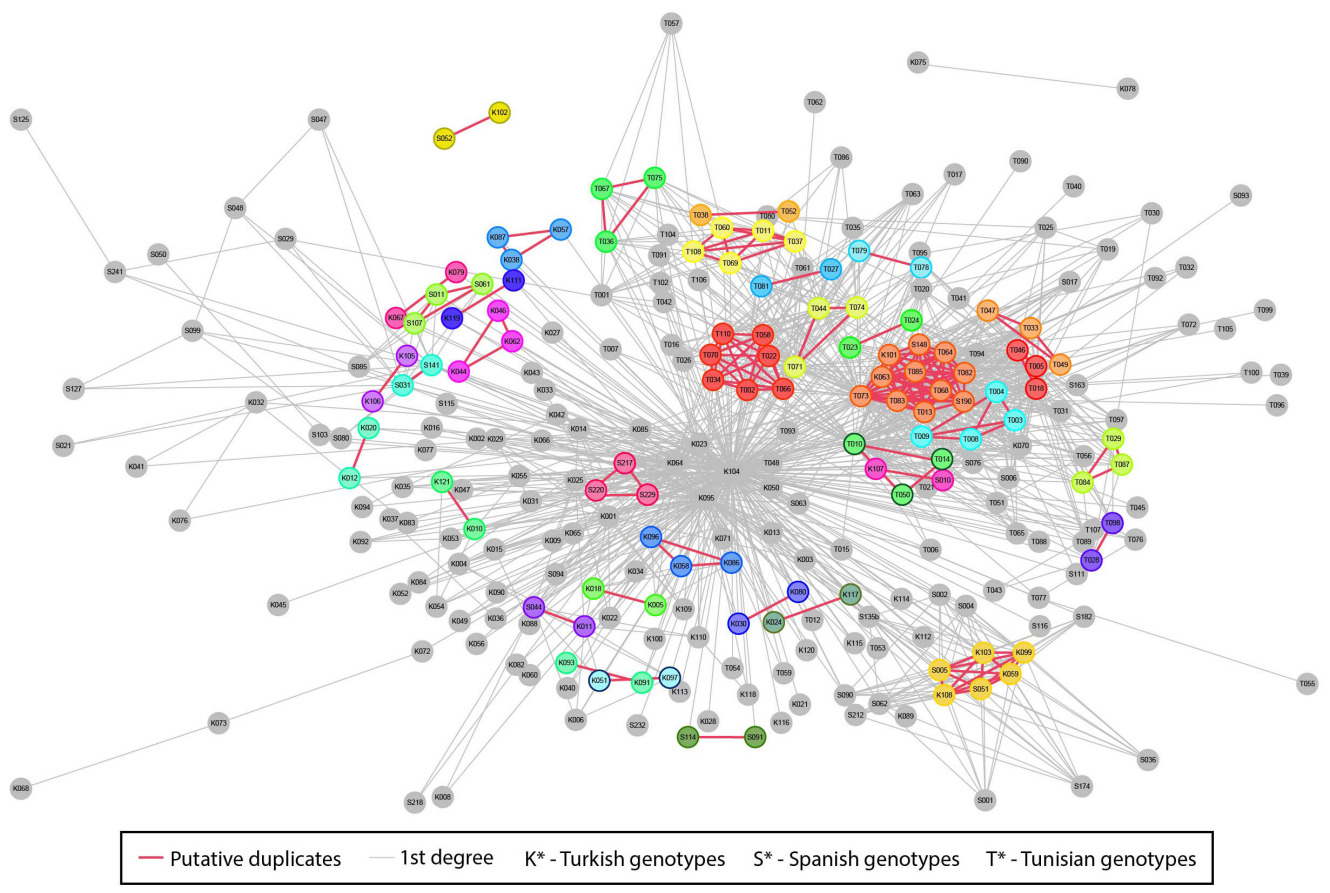
Network of first-degree relationship (grey) and possible duplicate (coloured) within and between the three germplasm banks. Node positions are computed using a weighted Kamada–Kawai layout. The length of each connection is proportional to the kinship value.

The patterns of identity-by-descent relationships were analysed among the 286 fig genotypes, providing insights into the relatedness among individuals within and across clusters and identifying cases of synonymy (possible duplicates) and 1^st^-degree, 2^nd^-degree, and 3^rd^-degree relatedness (**Supplementary Figure 6**; **Supplementary Table S12**). A network was constructed, including genotypes classified as possible duplicates and those with first-degree relationships. Possible duplicates, which had a kinship value ranging from 0.354 to 0.50, were identified as genetically highly closely related and highlighted with different colours (**Figure 5**). For example, in Spain, genotypes ‘Clon 300’ (S091) and ‘Granito’ (S114) showed genetic proximity with a high kinship value of 0.492.

Also, ‘Brocalet’ (S031) and ‘Bonjesusa’ (S141) (in light blue, on the left in **Figure 5**) showed genetic similarity (kinship value = 0.491), and although they showed phenotypic resemblance, they differed in productive type (PT), with ‘Brocalet’ classified as bifera type and ‘Bonjesusa’ as unifera type. A very similar SNP pattern was also found between more than two genotypes, as shown by ‘Panachée’ (S011), ‘Blanca R’ (S061), and ‘Burjassot V’ (S107) (in light green on the left of **Figure 5**), with kinship values of 0.492.

We also observed pairs of closely related genotypes from different collections. For example, the genotype ‘Smyrna’ (S044) of the Spanish collection was identified as genetically nearly identical to Turkish genotype ‘Sarilop-1029’ (K011) (in purple, bottom left in **Figure 5**, kinship value = 0.492). Genotypes ‘Kod-2 Nazareth’ (K107) and ‘Nazaret’ (S010) from the Turkish and Spanish collections were included in the cluster of Tunisian genotypes (**Figure 4C**) and showed high genetic similarity (pink, centre right in **Figure 5**, kinship value = 0.488). Within the cluster of Spanish genotypes, Turkish genotype ‘Kod-3 Banana’ (K102) (**Figure 4C**) showed close relatedness to Spanish genotype ‘Banane’ (S052) (in yellow, top left of **Figure 5**, kinship value = 0.491).

### 3.3 Genome-wide association study on plant and fruit traits of fig

**Figure 6:**
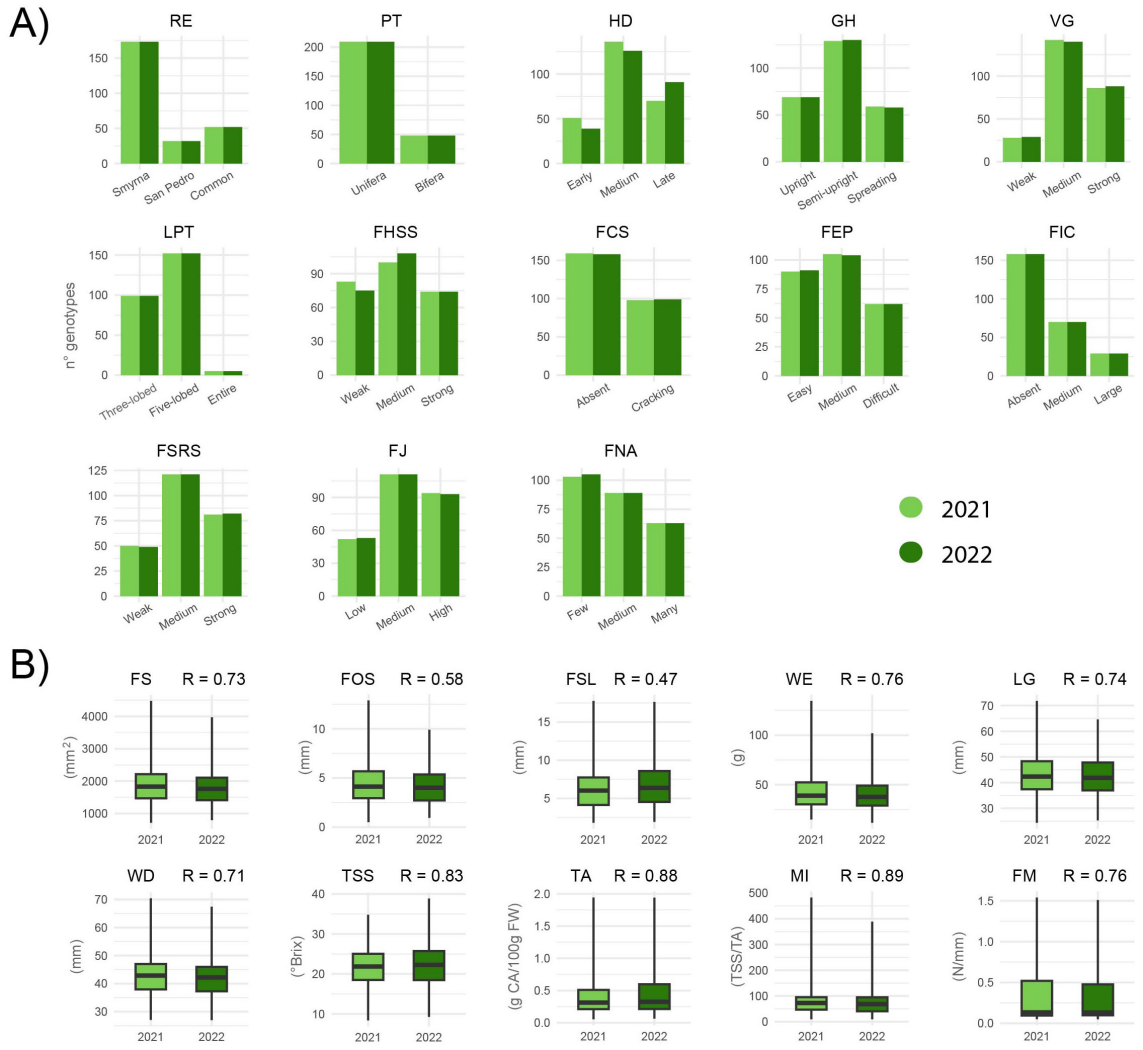
Analysis of 23 phenotypic traits in 2021 and 2022: **(A)** histograms of ordinal traits classified into categories; **(B)** estimated marginal means of quantitative traits. Phenotypic traits: reproduction (RE), productive type (PT), harvesting data (HD), growth habit (GH), vigour (VG), leaf predominant type (LPT), fruit attachment of stalk to stem (FHSS), fruit size (FS), fruit ostiole size (FOS), fruit stalk length (FSL), fruit cracking of skin (FCS), fruit ease of peeling (FEP), fruit internal cavity (FIC), fruit scratch resistance of skin (FSRS), fruit juiciness (FJ), fruit number of achenes (FNA), fruit weight (WE), fruit length (LG), fruit width (WD), total soluble solids (TSS), titratable acidity (TA), maturation index (MI), and firmness (FM). R indicates the estimated repeatability of quantitative trait.

The germplasm banks located in Spain, Turkey, and Tunisia provided an unprecedented overview of the diversity of plant and fruit traits of fig genotypes. Phenotypic characteristics were analysed on 257 of 286 fig genotypes in 2021 and 2022 (**Supplementary Table S13**). **Figure 6** illustrates the phenotypic variability occurring in our fig collections. Thirteen qualitative (**Figure 6A**) and 10 quantitative (**Figure 6B**) traits were measured. Repeatability was estimated for quantitative traits, ranging from 0.47 in fruit stalk length (FSL) to 0.89 in maturation index (MI) (**Figure 6B**, **Supplementary Table S14**).

The whole SNP dataset, phenotypic traits (**Supplementary Table S15**), and kinship matrix were used to perform GWAS. Bonferroni correction was applied to the GWAS results to reduce the likelihood of false positive associations, ensuring the identification of significant peaks. Manhattan plots, QQ-plots were generated for each trait, visualising the SNPs significantly associated with agro-morphological characteristics across *F. carica* chromosomes. GWAS revealed 481 significant SNPs associated with 11 fruit traits and productive type (PT) (**Supplementary Table S16**). Fruit traits included attachment of the stalk to stem (FHSS), internal cavity (FIC), juiciness (FJ), number of achenes (FNA), weight (WE), length (LG), width (WD), total soluble solid (TSS) content, titratable acidity (TA), maturation index (MI), and firmness (FM). For a subset of these SNPs, candidate genes were directly identified, as the SNPs were located within annotated gene models (**Supplementary Table S17**). When the significant SNPs did not fall within a gene, we defined LD window of 40,000 base pairs to search for putative candidate genes in the surrounding genomic region (**Supplementary Table S18**).

**Figure 7:**
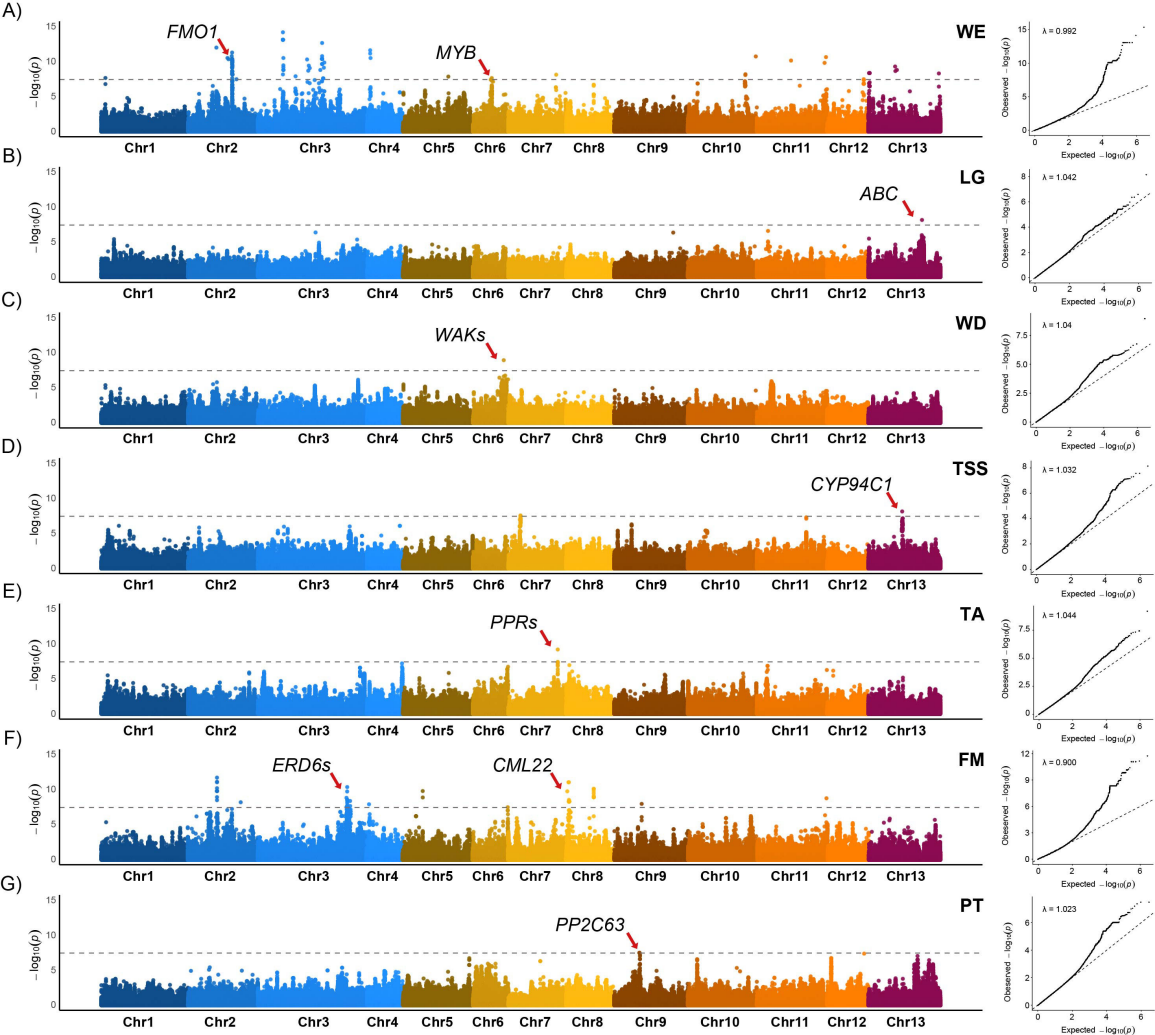
Manhattan plots of six fruit traits and productive type associated with GWAS: **(A)** fruit weight (FW); **(B)** fruit length (LG); **(C)** fruit width (WD); **(D)** total soluble solids (TSS) content; **(E)** titratable acidity (TA); **(F)** firmness (FM); **(G)** productive type (PT). The x-axis represents the 13 chromosomes of *Ficus carica*, and the y-axis shows the –log_10_(p-value). The grey horizontal line indicates the Bonferroni threshold, and the red arrows highlight the identified candidate genes. QQ-plots with the genomic inflation factor (λ) are shown alongside each Manhattan plot.

GWAS results were reported for traits of primary relevance to market and consumer preferences (**Figure 7**). In our study, 129 SNPs were associated with WE (**Figure 7A**). Among these, 21 SNPs were located within 15 genes (**Supplementary Table S17**). Six SNPs in chromosome 2 were located in a candidate gene, encoding a probable flavin-containing monooxygenase 1 (FMO1). The additive effect ( $\hat{a}$) of these loci was estimated at +5.61 g per copy of the alternative allele, corresponding to an expected difference of 11.2 g between homozygotes. Similarly, a single SNP on chromosome 6, located in a *MYB transcription factor* gene, showed a stronger additive effect ( $\hat{a}=+11.36$ g) (**Supplementary Table S16**). Trait distributions by genotype class for the top SNPs across traits are presented in **Supplementary Figure 7**. For some SNPs, a statistically significant dominance effect was observed.

We identified one SNP associated with LG on chromosome 13 and one SNP associated with WD on chromosome 6. The additive effect of the SNP on chromosome 13 was +9.73 mm per copy of the alternative allele. The SNP on chromosome 6 increased WD by +6.73 mm per copy of the alternative allele. Based on the position of these SNPs and LD interval, we identified one notable candidate gene potentially involved in LG, encoding an ABC transporter G family member 20 (**Figure 7B**). For the SNP associated with WD, we found two possible candidate genes, both encoding a wall-associated receptor kinase (WAK) (**Figure 7C**).

We detected three SNPs on chromosomes 7 and 13 associated with the TSS content (**Figure 7D**) and one SNP on chromosome 7 associated with TA (**Figure 7E**).

Regarding TSS, the SNP identified on chromosome 13 showed an additive effect of +3.66°Brix per copy of the alternative allele, with a candidate gene encoding cytochrome P450 94C1 (*CYP94C1*) located in the associated genomic region. For TA, the SNP on chromosome 7 was associated with an additive effect ( $\hat{a}=+0.27$ g citric acid/100 g fresh weight), with three candidate genes encoding pentatricopeptide repeat-containing proteins (PPRs) identified in the same region.

For FM, one SNP was identified within a potential candidate gene on chromosome 8, encoding a probable calcium-binding protein *CML22* (**Figure 7F**). The additive effect of this SNP was +0.63 N/mm. Other SNPs, located on chromosome 3, exhibited an additive effect of −0.31 N/mm, and two candidate genes encoding sugar transporters (ERD6-like 5 isoform X1) were identified.

Regarding the PT trait, a single significant SNP was found on chromosome 9. Within the LD interval of this SNP, we identified a probable protein phosphatase 2C 63 (*PP2C63*) gene (**Figure 7G**).

The GWAS results for the remaining traits are provided in **Supplementary Figures 8**, **9**; **Supplementary Tables 16**, **17**. Significant SNPs reported in **Supplementary Figure 8** are related to other morphological traits that are less interesting and relevant for the market and consumers but deserve further investigation to uncover novel genetic contributors to fruit traits in fig.

## 4 Discussion

In this study, we performed a comprehensive genome-wide analysis of genetic variations in fig trees from the Mediterranean region by examining 286 fig genotypes from Spanish, Turkish, and Tunisian collections. To our knowledge, this constitutes the most comprehensive assessment of genomic variation in cultivated fig to date, providing an extensive repository of genetic information, expanding on previous studies that mainly relied on SSR markers (Perez-Jiménez et al., 2012; Akin et al., 2021; Essid et al., 2021) or on smaller datasets combining SNPs and SVs (Bazakos et al., 2024). Our study builds upon these efforts by leveraging a substantially larger dataset of 286 fig genotypes, enabling a more comprehensive exploration of fig genome diversity.

### 4.1 Variant discovery and population diversification

High-quality WGR data were obtained for 286 fig genotypes from Mediterranean collections in Turkey (114 originating from Turkey and one from Israel), Spain (58 originating from Spain, 1 from Israel and 1 from Ethiopia), and Tunisia (110 originating from Tunisia).

We identified 1,374,111 high-quality SNPs across the 13 chromosomes of the *F. carica* genome, with over 700,000 found in genic regions. Most SNPs should have a low or moderate impact on gene function. The K_a_/K_s_ ratio was 1.2, slightly lower than that observed in a previous study in figs (1.35; Bazakos et al., 2024) and lower than that of other fruit trees, such as olive (1.45; Bazakos et al., 2023), mango (1.52; Wang et al., 2020), and sweet cherry (1.78; Xanthopoulou et al., 2020). In contrast, the fig K_a_/K_s_ was slightly higher than in peach (1.06; Li et al., 2019).

Analysis of InDels revealed 1,270,023 insertions and 1,178,743 deletions, of which 43.3% were found in genes (16.93% in introns and 2.25% in coding sequences) and adjacent sequences (12.98% upstream and 11.66% downstream). The most common were single nucleotide (53.64%), followed by dinucleotide (14.99%) and trinucleotide InDels (6.72%). Mirroring patterns have been observed in other fruit species, such as olive (Bazakos et al., 2023) and sweet cherry (Xanthopoulou et al., 2020).

Genome-wide CNV analysis revealed 94 CNGs and 121 CNLs, with 218 CNVs. Of these, 156 CNVs mapped 1227 genes. These genes contribute to various functions, encompassing acyltransferase activity and various organic- and dicarboxylic-acid transport processes. We also identified 1342 deletions and 21 duplications. Of these, we identified 606 genes affecting SVs, and they were involved in proteolytic processes and hydrolase activities.

Genes affected by CNVs suggest that these structural variations may contribute to environmental pressures by modulating the transport and cellular redistribution of organic acids and ions. These processes are crucial in cellular homeostasis processes, including pH regulation, carbon redistribution, osmotic adjustment, and signal transduction, particularly under stress conditions (Panchal et al., 2021; Wang et al., 2021). In contrast, genes affected by SVs are mainly associated with proteolytic functions, suggesting a role in protein turnover and regulated protein degradation. These structural variations may influence the plant’s capacity to respond to biotic and abiotic challenges by activating or removing specific proteins involved in stress signalling and degradation of damaged proteins (Luciński and Adamiec, 2023).

In our fig collection, the LD decay was rapid, around 18 kb. This is more comparable to fig cultivars from different geographic origins of the Mediterranean, where LD decay ranges from 8 to 15 kb (Bazakos et al., 2024) than to cultivars of other fruit tree species. *Prunus persica* (peach) and *Malus domestica* (apple) show much slower LD decay, with r² ≤ 0.2 observed between 800 and 1400 kb (Micheletti et al., 2015) and r² = 0.21 at 500 kb and 0.16 at 1000 kb, respectively (Kumar et al., 2013).

In population structure analysis performed using SNP data (**Figure 4**), the first two principal components explained 10% of the total genetic variability, which was similar to a previous study conducted on 53 genotypes from the Mediterranean region (Bazakos et al., 2024), in which the genetic variability explained by the first two components was approximately 15%. All population analyses showed the occurrence of three main clusters corresponding to the three countries of origin. However, there was evidence of intermixing, with some genotypes from Turkey, Spain, and Tunisia appearing in clusters associated with other countries. The FST (**Figure 4D**) showed moderate genetic differences between Spanish and Tunisian collections and between Turkish and Tunisian collections. Fewer differences were observed between Turkish and Spanish genotypes. These results confirmed the genetic diversity among genotypes from different national germplasm banks, highlighting the genetic diversity of fig germplasm in the Mediterranean region and confirming the patterns observed in a previous study that used a much smaller number of genotypes (Bazakos et al., 2024). In all three groups, H_0_ was slightly higher than H_e_, suggesting a slight deviation from Hardy–Weinberg equilibrium, due to cross-hybridisation or recent migration events among genotypes from the same group. The highest genetic variability was observed in the Turkish germplasm collection (*p* = 0.00178), followed by the Spanish (*p* = 0.00172) and Tunisian collections (*p* = 0.00168). SV-based analyses provided a complementary perspective on fig population structure. PCA performed using SVs clearly separated Tunisian genotypes. In contrast, Spanish and Turkish accessions showed partial overlap, a pattern consistent with lower genetic differentiation observed between these collections as indicated by FST values. Nevertheless, phylogenetic analysis based on SVs supported the presence of three main populations, in agreement with the clustering observed in SNP analysis (**Supplementary Figure 5**). Our results indicate that multiple datasets from different germplasm banks are needed to describe the genetic variability of *F. carica* in the Mediterranean region.

Kinship analysis resolved the cryptic relatedness among genotypes (**Figure 5**). For example, the ‘Smyrna’ genotype, which was included in the Turkish cluster, was identified as a duplicate of ‘1029-Sarilop’. ‘Sarilop’ is the most important Turkish variety, accounting for 90% of national production. This variety, prized for its suitability for drying, is characterised by a whitish-yellow colour, ideal moisture content (22–24%), and high sugar content (50–55%) (Nakilcioğlu and Hışıl, 2013). ‘Sarilop’ may have been included in Spanish collections under a different name, so it could be considered a synonym of ‘Smyrna’. Another example of genetic proximity was observed between ‘Clon 300’ and ‘Granito’. These two cultivars share the same SSR profile with 9 SSR markers, although they exhibit some distinct morphological features (Lopez-Corrales, personal communication). ‘Granito’ comes from Extremadura, whereas ‘Clon300’ comes from the Balearic Islands. Both are cultivated to the north of the Extremadura region for dried consumption. ‘Clon 300’ is likely a clone of ‘Granito’. Additionally, ‘Brocalet’ and ‘Bonjesusa’ showed genetic similarity, confirmed by analyses based on 9 SSR markers (Lopez-Corrales, personal communication). ‘Panachée’, ‘Blanca R’, and ‘Burjassot V’ showed similar SNP patterns. These three genotypes share 18 of 20 SSR markers, although only ‘Panachée’ has yellow and green bands on the fruit skin (Lopez-Corrales, personal communication). These data highlight the importance of molecular and morphological analyses in the classification of fruit tree varieties. Indeed, even a single mutation in a genotype could give rise to different phenotypic traits and thus to the creation of a new variety. We are conducting comparative analyses between ‘Panachée’, ‘Blanca R’, and ‘Burjassot V’ to understand which SNPs are correlated with the yellow and green bands of ‘Panachée’ fruit skin.

The similarity between genotypes may reflect the historical exchange of genetic material. These genotypes, originating from the same variety, may introduce silent mutations in their genome and be introduced in germplasm collections under different local names. Some examples are genotypes ‘Kod-2 Nazareth’ and ‘Nazaret’ from Turkish and Spanish collections, respectively, and ‘Kod-3 Banana’ from Turkey and ‘Banane’ from Spain. ‘Nazaret’ is a San Pedro type variety from Israel that is known for its high yield. It has probably been transferred to the Turkish and Spanish collections under two slightly different names. Similarly, ‘Banane’ is a peculiar variety with large fruit similar to a banana, also known as ‘Longue d’Aout’, and ‘Jérusalem’, which are widely grown in Europe and the USA. It was probably introduced into the Turkish and Spanish collections using two similar names.

Closely related fig genotypes have been reported in several studies documenting that sharing genotypes across countries, vegetative propagation, and local selection, are key factors shaping fig diversity (Achtak et al., 2010; Mazzeo et al., 2024; Radunić et al., 2025).

### 4.2 Genome-wide association studies

The phenotypic characterisation of *F. carica* genotypes across germplasm banks in Spain, Turkey, and Tunisia highlights the significant biological diversity shaped by genetic and/or environmental factors. Repeatability estimates on quantitative traits highlighted different sensitivities to environmental variations. Traits such as FSL and FOS showed lower repeatability (R = 0.47 – 0.58), indicating a higher sensitivity to environmental effects. Biochemical traits, such as total soluble solids and titratable acidity, are known to be strongly influenced by environmental conditions, including temperature, water availability, and harvesting time. Nevertheless, repeatability estimates obtained from replicated measurements were high for both traits (TSS R = 0.83; TA R = 0.88), suggesting a predominant genetic contribution in the studied germplasm. These results indicate that, although environmental effects are present, genotypic differences are robust and reproducible across replicates, supporting the use of these traits for genetic analyses and association with structural variants.

Drawing on the comprehensive phenotypic and genomic data collected, GWAS was performed to identify links between genetic and phenotypic variation in relation to traits considered most relevant for market and consumer preferences (**Figure 7**).

The association analysis presented in this study has some intrinsic limitations. The genotypes included in the panel were not replicated across multiple environments, and therefore phenotypic measurements might partially reflect environmental effects in addition to the underlying genetic component. Moreover, the plants differed in age, a factor that can also influence fruit-related traits in perennial fruit trees (Pang et al., 2024). As a consequence, a formal analysis of genotype × environment interactions could not be performed. Nevertheless, this limitation is common in studies dealing with perennial fruit crops, and particularly with minor or underutilized species, for which replicated multi-environment trials are often not available (Zadokar et al., 2025). Despite these constraints, the large number of genotypes analyzed, the wide genetic diversity represented, and the consistency of some associations with known biological expectations suggest that the results provide a useful insight into the genetic architecture of fruit traits in fig.

GWAS identified SNPs and candidate genes associated with traits of market relevance influencing pricing, sales, and consumer preferences, such as WE, LG, WD, FM, TSS content, and TA (Aksoy et al., 1992; Aljane and Ferchichi, 2007; Flaishman et al., 2007; Çalişkan and Polat, 2008; Crisosto et al., 2010; Trad et al., 2013; Mahmoudi et al., 2018). Among the candidate genes identified by GWAS, the *FMO1* gene emerges as potentially influencing WE. Flavin-containing monooxygenases (FMOs) are a class of flavoenzymes implicated in auxin biosynthesis and glucosinolate metabolism (Wang et al., 2023a). Auxin is a critical hormone that shapes fruit initiation and growth; therefore, variation in this gene could influence fruit expansion and the final weight. Interestingly, a mutation in the FMO1 gene in tomato leads to reduced WE and size (Wang et al., 2023b), whereas in our study, specific alleles of FMO1 were associated with increased WE (+5.61 g), suggesting these variants may represent favourable alleles. Another candidate gene possibly involved in WE encodes an *MYB* transcription factor. The MYB family of transcription factors is characterised by a conserved domain that enables them to bind to DNA. MYB proteins in plants regulate diverse pathways, including secondary metabolism (such as the anthocyanin biosynthesis pathway), development, signal transduction, and disease resistance (Ambawat et al., 2013). Previous studies have demonstrated that MYB genes activate anthocyanin pigmentation in the skin, flesh, and foliage of apple (Espley et al., 2007; Allan et al., 2008) as well as mangosteen (Palapol et al., 2009) and regulate flavonoid biosynthesis in peach (Ravaglia et al., 2013). Furthermore, a recent study by Zhang et al. (2024a) revealed that MYBs negatively regulate fruit ripening in citrus and tomato and modulate fruit size in citrus. Consistently, certain alleles of this MYB gene were also associated with increased WE (+11.36 g), indicating that they may be favourable variants for improving WE.

LG and WD influence pricing and sales, with larger, well-proportioned fruit often perceived to be of higher quality and desirable. Concerning LG, the *ABC* (*ATP-Binding Cassette*) *transporter G family member 20* was identified as a candidate gene. ABC transporters are proteins involved in transporting a wide range of molecules, including organic acids, metal ions, phytohormones, and secondary metabolites. These transporters play an essential role in plant growth and development, particularly in fruit development, as observed in cedar (Zhang et al., 2024b), tomato (Ofori et al., 2018; Tao et al., 2024), and strawberry (Shi et al., 2020). For WD, two *WAK* genes were identified. WAKs belong to a subfamily of receptor-like kinases linked to the cell wall and are thought to function as sensors of the extracellular environment, initiating intracellular signalling pathways. In tomato, WAK-encoding genes have been observed to exhibit distinct expression patterns during various fruit development and ripening stages, with higher expression during the fruit expansion phase and a decline as the fruit ripens (Sun et al., 2020). Although the associated SNPs were not located within genes, the alleles at these loci were linked to increased LG and WD. Their proximity to ABC transporter and WAK genes, both involved in cell expansion and fruit growth, suggests a possible functional relationship. These findings suggest that WAK and ABC transporter genes may play a similar role in regulating cell expansion and fruit development in fig, making them promising candidates for further functional analyses.

The TSS content and TA are also important traits linked to consumer preferences, as they significantly influence the taste of fruit. For TSS, an association was detected on chromosome 13, where allelic variation corresponds to an increase of +3.66°Brix per copy of the alternative allele. The associated region included a gene encoding cytochrome P450 94C1 (CYP94C1). Cytochrome P450 enzymes (CYPs) form a large and evolutionarily conserved superfamily involved in several oxidative reactions in plant metabolism, and they play a crucial role in hormone biosynthesis and degradation, affecting key developmental processes (Hansen et al., 2021). Several CYP genes, including *CYP94C1*, show dynamic expression patterns during early fruit development, contributing to hormonal regulation, cell wall remodelling, and defence mechanisms. The expression of many CYP genes declines as fruit matures, reflecting a shift in metabolic priorities (Minerdi and Sabbatini, 2024). In tomato, *CYP90B3*, a member of a different CYP subfamily, catalyses a key step in brassinosteroid biosynthesis. Its expression increases during ripening and is linked to fruit softening, sugar accumulation, and enhanced flavour (Hu et al., 2020). Although belonging to distinct subfamilies, such CYPs may function sequentially or co-ordinately within shared metabolic pathways. Despite the apparent involvement of CYP94C1 in early fruit development, its role in *F. carica* remains unexplored, representing a promising target for future research into fruit quality traits, such as TSS.

For TA, a SNP on chromosome 7 was linked to an additive effect of +0.27 g citric acid/100 g fresh weight. The associated SNP was close to three genes encoding PPRs. *PPR* genes belong to one of the largest gene families in plants and encode important RNA-binding proteins involved in the regulation of plant growth and development by influencing the expression of organellar mRNA transcripts (Subburaj et al., 2020). Molecular evidence highlights the significant roles of *PPR* genes in regulating fruit development, ripening, and flesh coloration, as demonstrated in crops, such as tomato, melon, and watermelon (Eriksson et al., 2004; Pesaresi et al., 2014; Galpaz et al., 2018; Subburaj et al., 2020). These findings suggest that *PPR* genes may also contribute to other key fruit quality traits, including those related to TA in fig fruit.

FM is a valuable attribute for selecting fruit in optimum condition for harvesting and contributes to extending the shelf life of fruit. The strongest association was observed on chromosome 8, with the alternative allele increasing FM by +0.63 N/mm. This SNP occurred within a gene encoding a probable calcium-binding protein *CML22*. Calcium ions play a role in various developmental and adaptive processes in plants as secondary messengers. Calmodulin-like (CML) proteins act as primary Ca^2+^ sensors and regulate diverse cellular functions (La Verde et al., 2018). Ding et al. (2018) highlighted the roles of *CML* gene families in fruit development, temperature stress responses, and the ripening process in papaya. In contrast, loci on chromosome 3 were associated with decreased FM (−0.31 N/mm) and were situated near two genes encoding sugar transporters ERD6-like 5 isoform X1. These proteins regulate glucose transport from the vacuole to the cytosol, playing a key role in cellular sugar balance, tissue turgor, and stress response. These transporters are conserved across multiple fruit species and contribute to sugar accumulation in mature fruit (in citrus, grape, apple, and pear) by influencing vacuolar sugar dynamics, which influences FM (Nishio et al., 2021).

PT are classified as unifera and bifera, i.e., characterised by one or two fig productions per year. A candidate gene involved in determining PT encodes a probable protein phosphatase 2C 63. Members of the protein phosphatase 2C (PP2C) group are negative modulators of protein kinase pathways activated by diverse environmental stress conditions or developmental signalling cascades, for example, within the abscisic acid (ABA)-mediated signalling network. Ectopic expression of *FsPP2C2* in Arabidopsis made the plants more sensitive to ABA and abiotic stress in both seeds and vegetative tissues, delaying flowering (Reyes et al., 2006). The identification of this gene in this study suggests a potential role in regulating the unifera/bifera fruiting habit, likely through the modulation of ABA-mediated developmental pathways.

In addition to the described candidate genes with known functions related to fruit traits, we identified a broader set of loci associated with different traits. Some of these loci do not overlap with annotated genes but probably harbour novel regulators of fruit development and quality. These additional associations, detailed in **Supplementary Figure 8**; **Supplementary Tables 16**, **17**, represent promising targets for future functional validation and may provide new insights into the genetic basis of key agronomic traits in fig.

## 5 Conclusion

This study presents one of the most comprehensive genomic and phenotypic analyses conducted to date on *F. carica* L., based on a panel of 286 genotypes collected from three Mediterranean countries. Through high-density SNP genotyping and detailed phenotypic evaluations, we provided valuable insights into the genetic diversity, population structure, and trait architecture of fig cultivars. The results enabled the identification of possible cases of synonymies and the estimation of genetic diversity among genotypes, contributing to the management of genetic resources in germplasm banks. GWAS revealed numerous marker–trait associations and candidate genes, some of which carried favourable alleles underlying important agro-morphological characteristics, offering promising tools for fig marker-assisted selection and genome editing. These findings lay a solid foundation for future breeding efforts aimed at improving fruit quality, yield, and adaptability, and for developing regional and global catalogues to enhance the characterisation, conservation, and sustainable use of fig biodiversity in the face of evolving agricultural challenges.

## Data Availability

The raw sequencing data have been deposited in the National Center for Biotechnology Information (NCBI) Short Read Archive (SRA) database under accession number PRJNA1289403.
