# Supplementary material for: Exploring the genetic diversity of Mediterranean fig trees highlights genes associated with fruit traits
Source: Front Plant Sci. 2026 Feb 10;17:1750632. doi: 10.3389/fpls.2026.1750632 (PMC12929547; doi:10.3389/fpls.2026.1750632)
Supplement: Supplementary file 1 [file DataSheet1.docx]

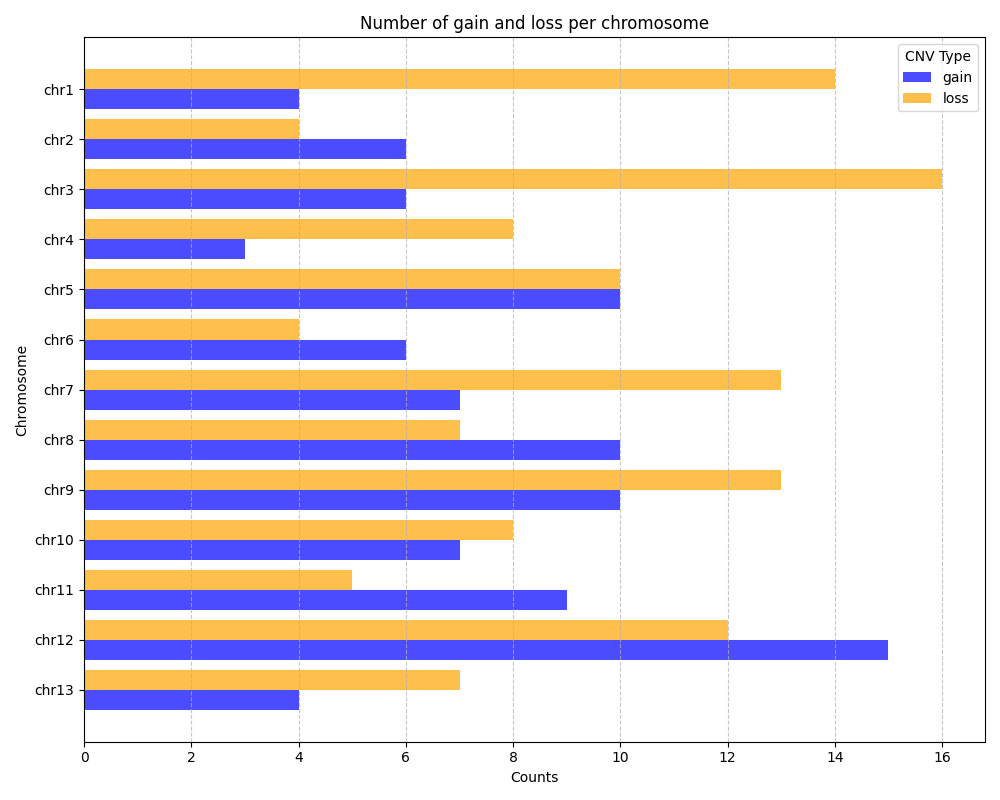


Supplementary Figure 1. Distribution of CNVs across genotypes. In yellow the copy number of losses, in blue the copy number of gains.


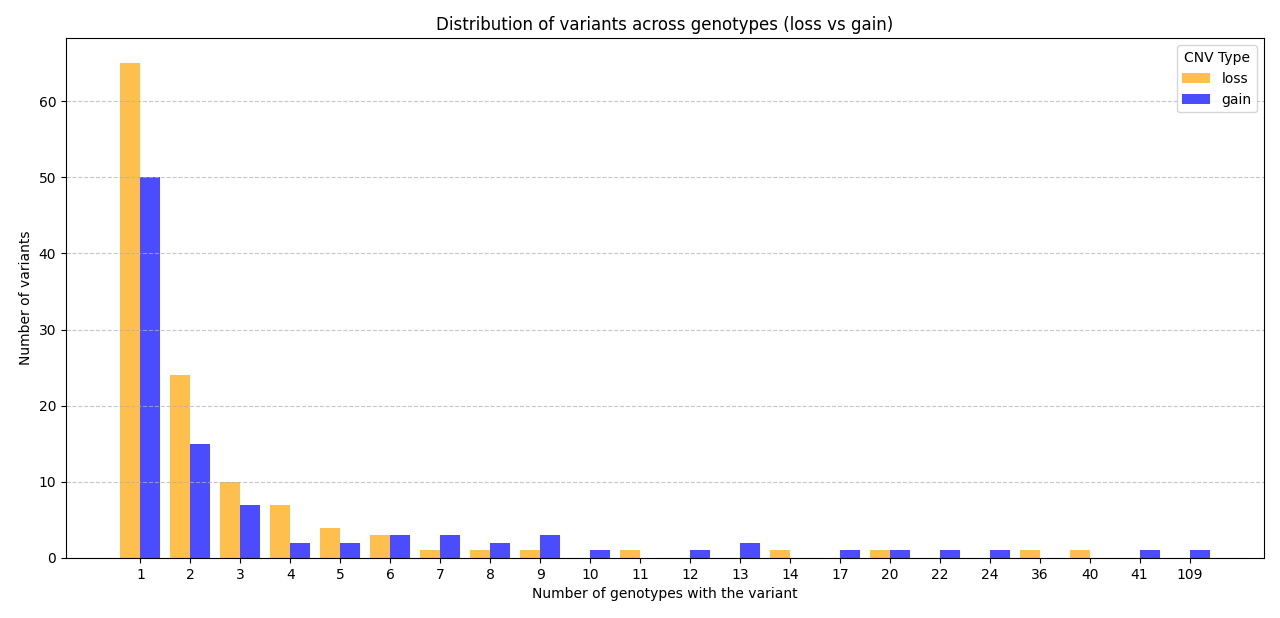


Supplementary Figure 2. Number of CNVs for each chromosome. In yellow the copy number of losses, in blue the copy number of gains.


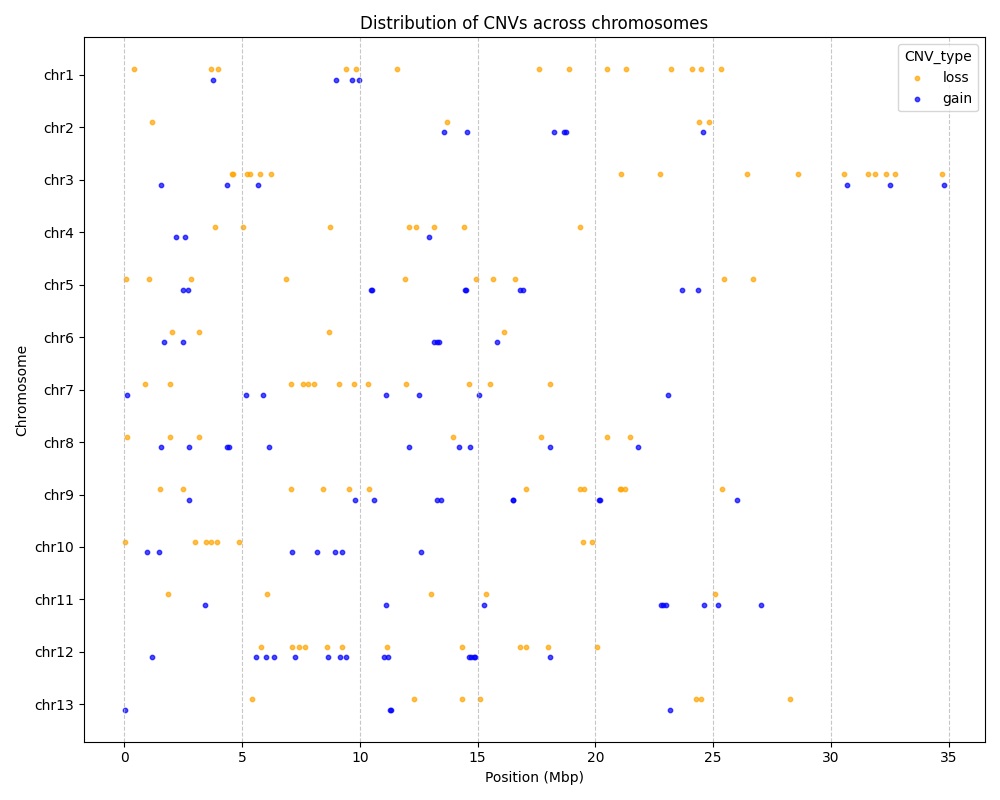


Supplementary Figure 3. Genome-wide distribution of CNVs. In yellow the copy number of losses, in blue the copy number of gains.


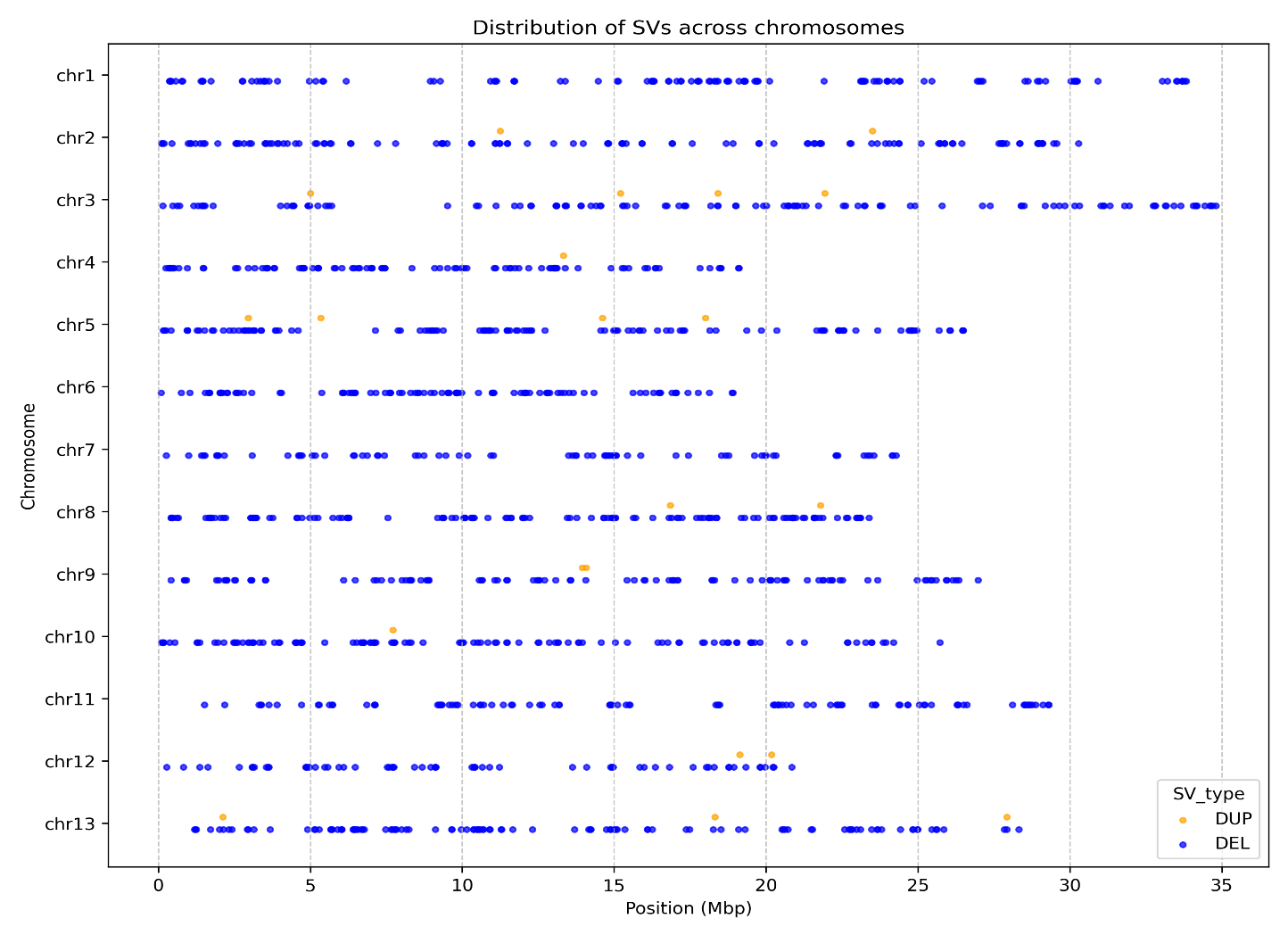


Supplementary Figure 4. Genome-wide distribution of SVs variants. In yellow duplications and in blue deletions.


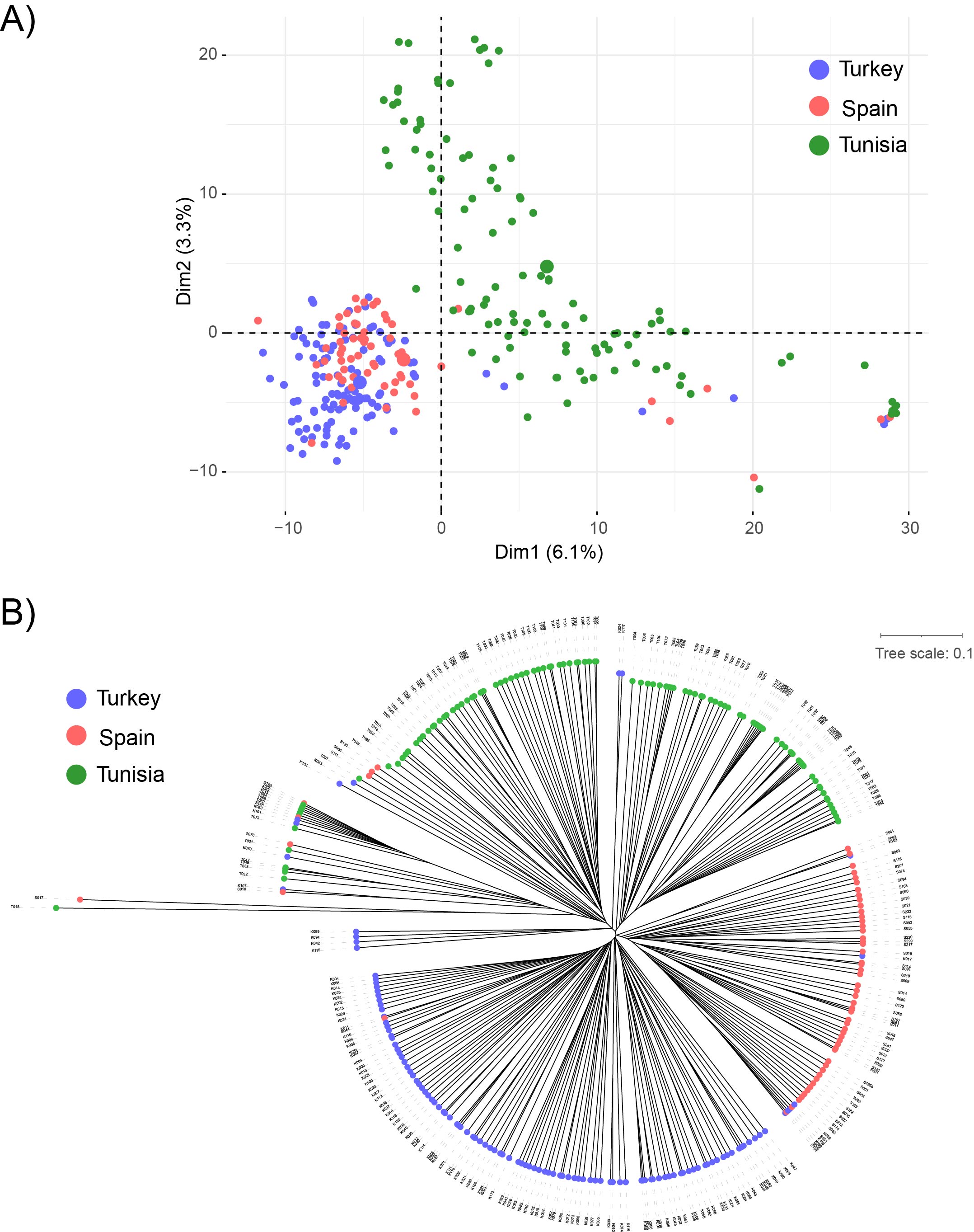


Supplementary Figure 5. Analysis of genetic diversity among fig genotypes based on structural variants (SVs). (A) Principal component analysis (PCA) showing the projection of the first two axes. (B) Neighbor-joining dendrogram, with the scale bar indicating genetic distance.


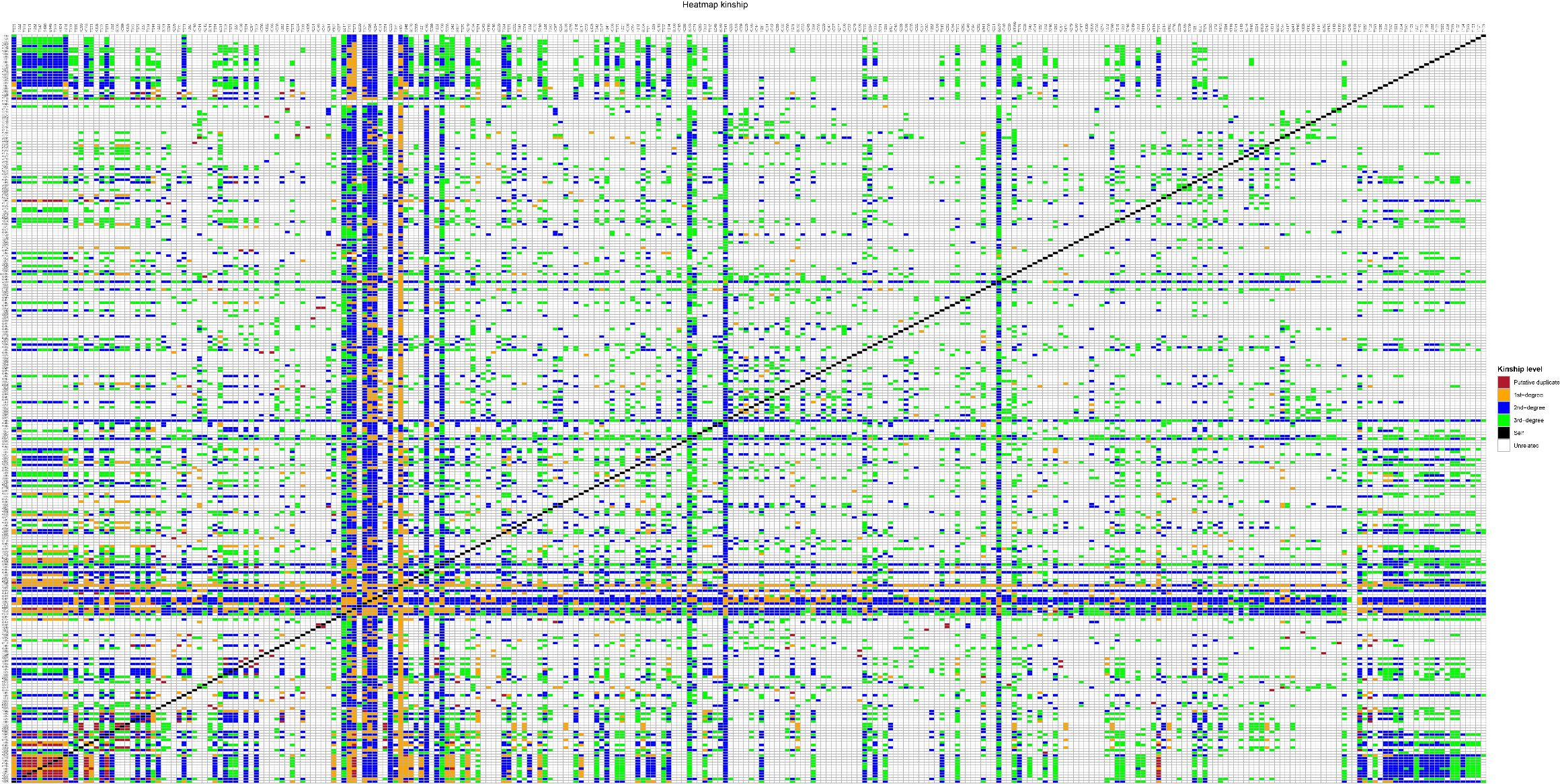


Supplementary Figure 6. Heatmap of case of synonymies (possible duplicate), 1^st^-degree, 2^nd^-degree, 3^rd^-degree of relatedness among the 286 fig tree genotypes.


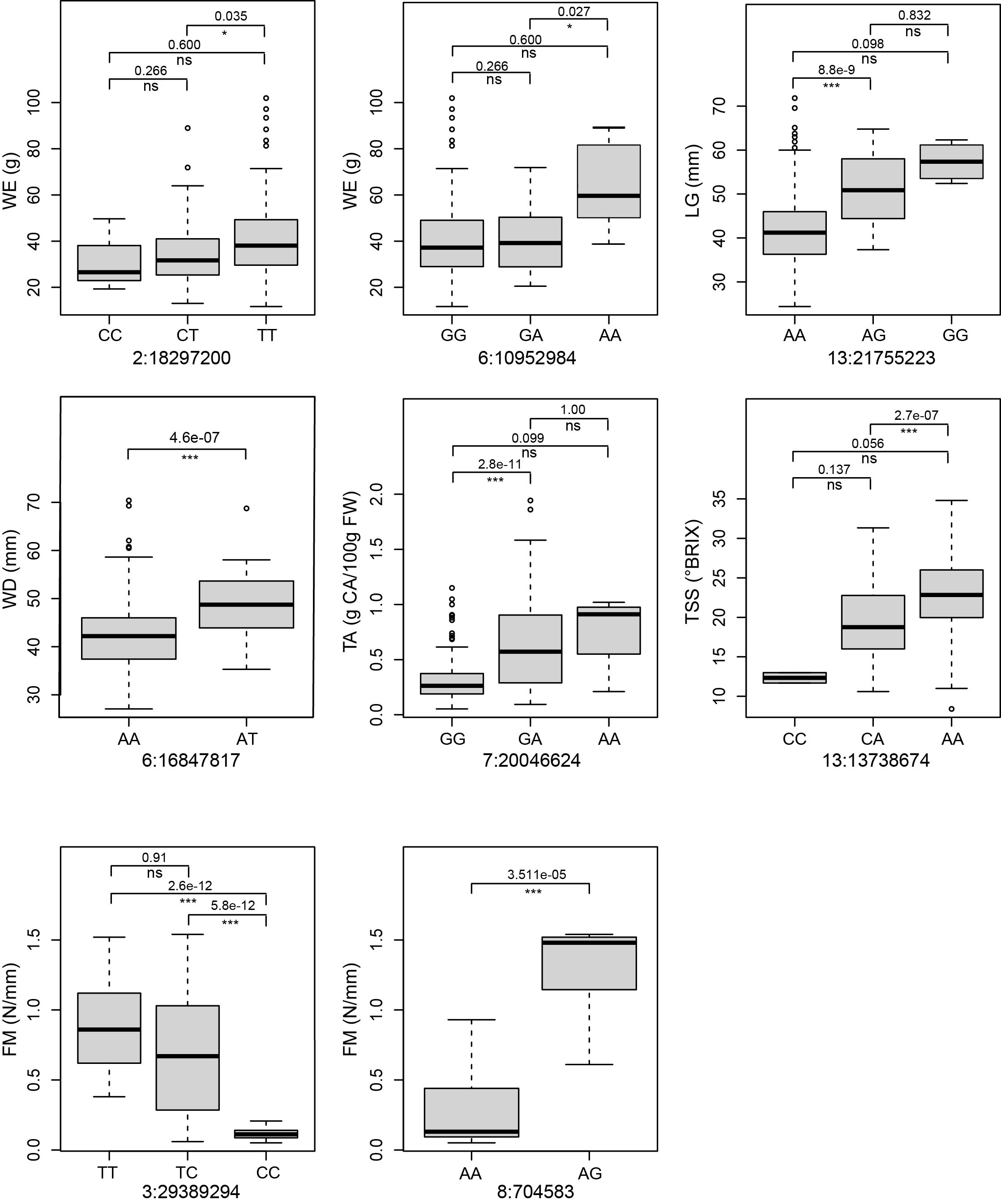


Supplementary Figure 7. Phenotypic trait values stratified by genotype for the top-associated SNPs across discussed traits. Each panel shows the distribution of trait values (fruit weight (WE), fruit length (LG), fruit width (WD), total soluble solids (TSS), titratable acidity (TA), firmness (FM)) for the different genotype classes of the corresponding SNP. The SNP ID is indicated below each distribution. Differences between genotype classes were assessed using Wilcoxon tests; both the exact *p*-values and significance levels indicated by asterisks (* *p* < 0.05, ** *p* < 0.01, *** *p* < 0.001) are shown in the figure.


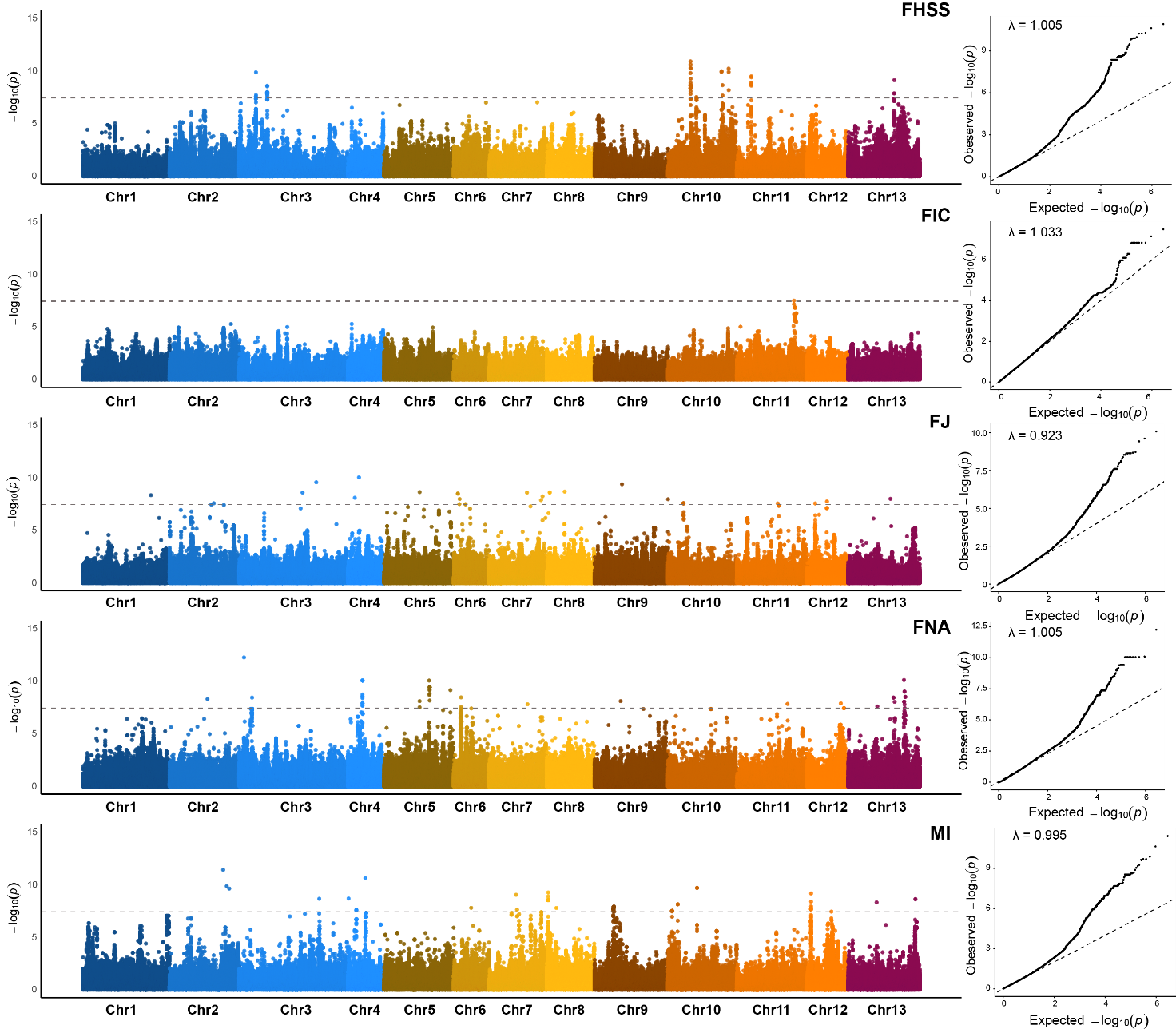


Supplementary Figure 8. Genome-wide association results for six additional traits with significant associations. Manhattan plot and QQ-plot of Fruit attachment stalk to stem (FHSS), Fruit internal cavity (FIC), fruit juiciness (FJ), fruit number of achenes (FNA), and maturation index (MI). Above each QQ-plot is reported the genomic inflation factor (λ) of each trait.


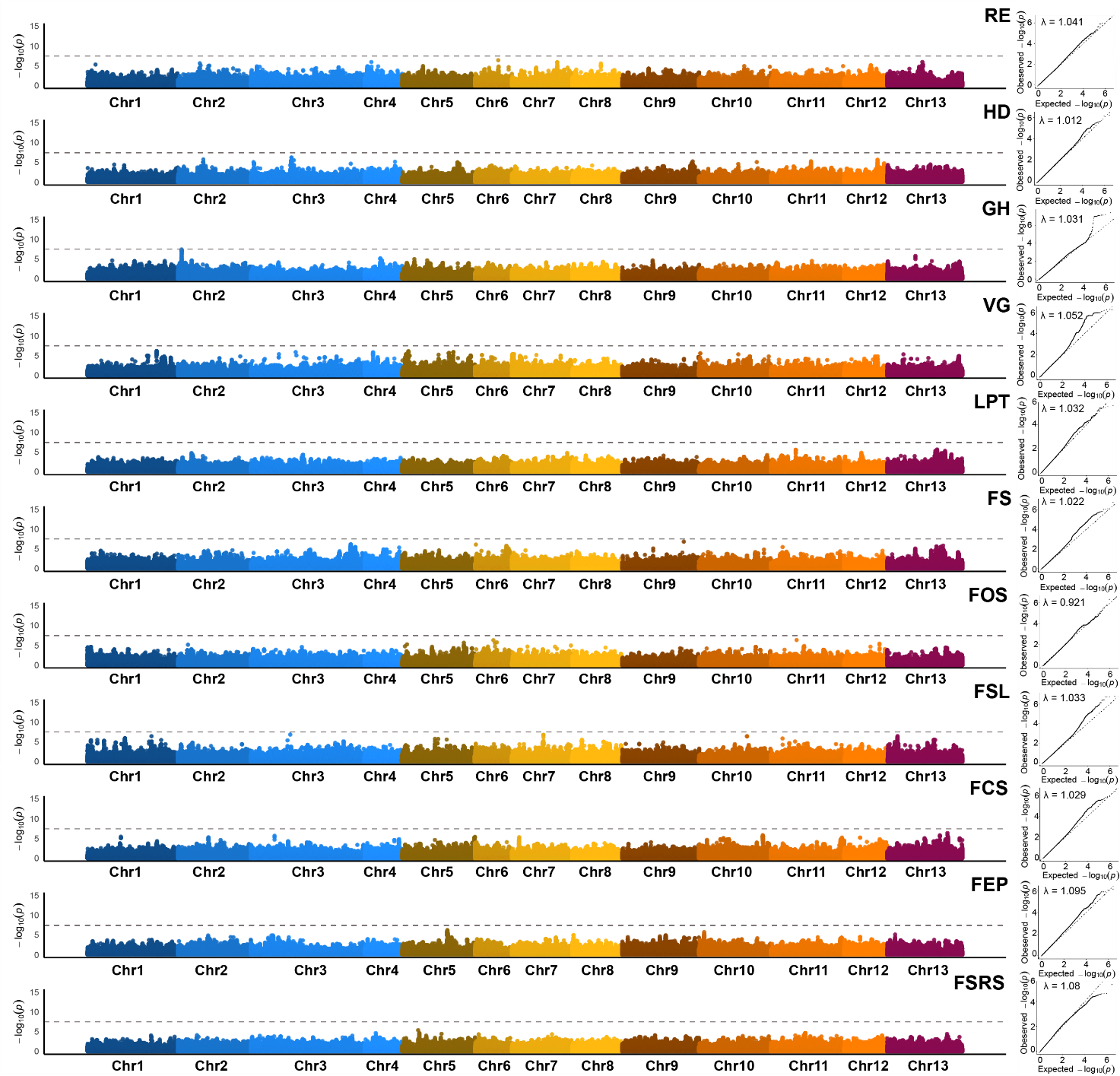


Supplementary Figure 9. Summary of non-significant GWAS results. Manhattan plot and QQ-plot of Reproduction (RE), harvesting date (HD), growth habit (GH), vigor (VG), leaf predominant type (LPT), fruit size (FS), fruit ostiole size (FOS), fruit stalk length (FSL), fruit cracking of skin (FCS) fruit easy of peeling (FEP), and fruit scratch resistance of skin (FSRS). Above each QQ-plot is reported the genomic inflation factor (λ) of each trait.
